# Ionic Combisomes: A New Class of Biomimetic Vesicles to Fuse with Life

**DOI:** 10.1002/advs.202200617

**Published:** 2022-04-07

**Authors:** Anna M. Wagner, Jonas Quandt, Dominik Söder, Manuela Garay‐Sarmiento, Anton Joseph, Vladislav S. Petrovskii, Lena Witzdam, Thomas Hammoor, Philipp Steitz, Tamás Haraszti, Igor I. Potemkin, Nina Yu. Kostina, Andreas Herrmann, Cesar Rodriguez‐Emmenegger

**Affiliations:** ^1^ DWI – Leibniz Institute for Interactive Materials Forckenbeckstraße 50 Aachen 52074 Germany; ^2^ Institute of Technical and Macromolecular Chemistry RWTH Aachen University Worringerweg 2 Aachen 52074 Germany; ^3^ Chair of Biotechnology RWTH Aachen University Worringerweg 3 Aachen 52074 Germany; ^4^ Physics Department Lomonosov Moscow State University Leninskie Gory 1–2 Moscow 119991 Russian Federation; ^5^ National Research, South Ural State University Chelyabinsk 454080 Russian Federation; ^6^ Institute for Bioengineering of Catalonia (IBEC) Carrer de Baldiri Reixac, 10, 12 Barcelona 08028 Spain; ^7^ Institució Catalana de Recerca i Estudis Avançats (ICREA) Passeig Lluís Companys 23 Barcelona 08010 Spain

**Keywords:** amphiphilic comb polymers, bottom‐up synthetic biology, hybrid vesicles, polyelectrolyte‐surfactant complexes, polymersomes, synthetic biomembranes, vesicle fusion

## Abstract

The construction of biomembranes that faithfully capture the properties and dynamic functions of cell membranes remains a challenge in the development of synthetic cells and their application. Here a new concept for synthetic cell membranes based on the self‐assembly of amphiphilic comb polymers into vesicles, termed ionic combisomes (i‐combisomes) is introduced. These combs consist of a polyzwitterionic backbone to which hydrophobic tails are linked by electrostatic interactions. Using a range of microscopies and molecular simulations, the self‐assembly of a library of combs in water is screened. It is discovered that the hydrophobic tails form the membrane's core and force the backbone into a rod conformation with nematic‐like ordering confined to the interface with water. This particular organization resulted in membranes that combine the stability of classic polymersomes with the biomimetic thickness, flexibility, and lateral mobility of liposomes. Such unparalleled matching of biophysical properties and the ability to locally reconfigure the molecular topology of its constituents enable the harboring of functional components of natural membranes and fusion with living bacteria to “hijack” their periphery. This provides an almost inexhaustible palette to design the chemical and biological makeup of the i‐combisomes membrane resulting in a powerful platform for fundamental studies and technological applications.

## Graphical abstract

Comb polymers with a zwitterionic backbone and ionically‐linked side chains are self‐assembled into a new class of vesicles that amalgamates the high stability of polymersomes with the biomimetic thickness, flexibility, and lateral mobility of liposomes. The faithful biomimicry enables to integrate functional components and hijack the complex periphery of living cells by fusion.

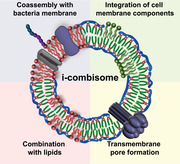

## 1 Introduction

Bottom‐up synthetic biology aims at building living cells by assembling nonliving modules into microcompartments displaying functionality and adaptivity as found in natural cells.^[^ ^1^ ^]^ Such a constructivist approach can reduce the cellular complexity within the grasp of contemporary science. This paradigm holds promise to unveil some of the most daunting questions: related to the origin of life, the transition from inanimate to living, and the emergence of diseases.^[^ ^1^, ^2^ ^]^ Moreover, not being constrained to simply copy the current life forms enables to engineer synthetic cells with biologically‐inspired but augmented or even completely new functions to open new horizons for biomedicine, sensing, and therapeutics.

Arguably, biological membranes are one of the central elements of natural cells. They are formed by the self‐assembly of lipids, proteins, and sugars giving rise to a multiplicity of functions.^[^ ^3^ ^]^ They give shape and volume to cells, provide compartmentalization of chemical components and allow sustaining chemical and energy gradients across them, which is a sine qua non condition for the existence of life. Through them, cells feed, grow, and communicate with their exterior. Such enormous richness of functions is achieved not only by the chemistry of the constituent elements but mainly by their dynamic topological organization driven by ultraweak interactions.^[^ ^4^ ^]^ Mimicking all the functions of the membrane is an insurmountable task, thus efforts have been directed at developing systems that recapitulate “some” functions using simple building blocks. The majority of works on biomimetic cell membranes exploited the self‐assembly of either lipids, amphiphilic block copolymers, or Janus dendrimers into liposomes, polymersomes, and dendrimersomes.^[^ ^5^ ^]^ Each of the families of amphiphiles has distinct advantages, which have led to highly specific uses. Liposomes display flexibility and lateral mobility close to natural membranes and have a thickness perfectly matching the one of their living counterparts.^[^ ^2^, ^6^ ^]^ This ensures that they can be readily functionalized with membrane proteins.^[^ ^7^ ^]^ However, their limited mechanical, thermal, and chemical stability as well as their intrinsic dispersity in size and shape preclude some advanced functions.^[^ ^2^, ^7^, ^8^ ^]^ The problem of stability is solved by polymersomes. Their higher molecular weight and concomitant entanglement of the hydrophobic blocks result in improved mechanical stability (energy at break).^[^ ^4^, ^9^ ^]^ Moreover, it allows to design and control the surface topology to generate reactive nanodomains that mimic rafts.^[^ ^10^ ^]^ But the higher molecular weights come at the cost of thicker, less flexible membranes with lower lateral mobility. This significantly hampers the insertion of transmembrane proteins, the mixing with functional lipids, and the mimicry of functions that demand remodeling of the membrane, such as engulfment, exocytosis, division, fusion, etc.^[^ ^9^, ^11^ ^]^ Most of these disadvantages have been alleviated with graft copolymers consisting of highly flexible, low molecular weight poly(dimethyl siloxane) backbones (PDMS), to which poly(ethylene oxide) (PEO) is sparingly grafted.^[^ ^12^ ^]^ The low density of grafts (one every13 dimethylsiloxane units) allows the backbone to bend, generating a liquid‐like hydrophobic core covered by PEO‐mushrooms and a lamellar lyotropic phase.^[^ ^12h^ ^]^ These special polymersomes display membranes with biomimetic thickness and dynamics, allowing the incorporation of transmembrane proteins as well as the coassembly with lipids.^[^ ^12c‐e^ ^]^ However, a more detailed examination revealed the formation of nano and microdomains of lipids^[^ ^13^ ^]^ followed by phase separation and fission into liposome and polymersome daughter vesicles.^[^ ^12^, ^14^ ^]^ Dendrimersomes are the most recent biomembrane system in which Janus dendrimers provide a xenobiotic surrogate for lipids and glycolipids.^[^ ^15^ ^]^ They display superior mechanical stability compared to liposomes with energies at break close to polymersomes, while maintaining the thickness, flexibility, and permeability close to the one observed for natural cell membranes.^[^ ^15^, ^16^ ^]^ They have been designed to fully or partially mix with lipids, readily incorporate proteins, drugs, nucleic acids, and form hybrids with bacteria and eukaryotic cells.^[^ ^17^ ^]^ Besides, they are used as a tool to dissect how the display of glycan nanoarrays controls biological activity and to perform basic cellular functions such as endocytosis.^[^ ^18^ ^]^ However, the required synthetic efforts have precluded their widespread use.

**Figure 1:**
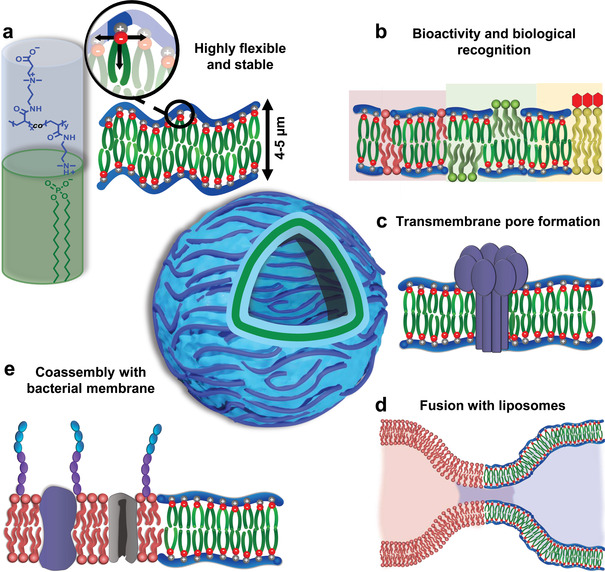
i‐Combisomes concept: iCPs self‐assembled into biomimetic unilamellar vesicles where the backbone is a rod confined at the water interface. a) The chemical structure of iCP shows the hydrophilic backbone of poly(CBAA‐*co*‐DMAPAA) to which the hydrophobic DDP is complexed. In water, the iCPs form bilayers with biomimetic thickness and high flexibility. b) Biofunctionalization of the i‐combisome membrane with lipids and generation of raft‐like domains. c) Formation of a pore by the insertion of pore‐forming peptides. d) Facile fusion with liposomes. e) Formation of hybrid protocells by fusion with living bacteria.

Herein, we introduce a novel concept for synthetic cell membranes based on the self‐assembly of ionically‐linked comb polymers (iCPs) into vesicles referred to as i‐combisomes. The iCPs consist of a hydrophilic backbone of poly(carboxybetaine acrylamide‐*co*‐*N*,*N*‐dimethylaminopropyl acrylamide) (poly(CBAA‐*co*‐DMAPAA)) to which lipid‐like hydrophobic side tails are appended by the complexation of didodecylhydrogen phosphate (DDP) with the free amines of DMAPAA (**Figure** **1**). The iCPs belong to the family of polyelectrolyte‐surfactant complexes with liquid crystalline behavior.^[^ ^19^ ^]^ Using a combination of atomistic molecular dynamic simulations, optical, electron, and force microscopies we screened a library of iCPs with tailored structural variations to unravel the structure and properties of the i‐combisomes. The high density of alky tails in the iCPs drives the formation of the hydrophobic core of the membrane and forces the hydrophilic backbone into a rod conformation confined to two dimensions at the water‐bilayer interface. Such organization results in membranes having a biomimetic thickness that is independent of the molecular weight of the amphiphile, in drastic contrast to polymersomes and block copolymers. Moreover, the noncovalent coupling of the hydrophilic and hydrophobic parts and the nonentangled nature of the hydrophobic domains resulted in more degrees of freedom, which manifested in membranes that amalgamated the best properties of liposomes and polymersomes. The i‐combisomes are stable up to 80 °C while displaying a lateral mobility and flexibility unprecedented for macromolecular amphiphiles, but closely resembling the dynamics in liposomes.

The i‐combisomes offer a molecular periphery suitable for chemical and physical functionalization for augmented functions. We showed that their high level of biomimicry enabled the integration of components of natural membranes, such as pore‐forming proteins, structure‐directing phospholipids, glycolipids, and nucleic acids. Furthermore, we demonstrated that the i‐combisomes could readily fuse with vesicles without the need for external energy supply or fusion mediators such as SNARE or amphiphiles with a net charge. The fusion with living bacteria enables to “hijack” a complex molecular periphery to endow the hybrid synthetic cells with some biological functions of natural membranes. Finally, we proposed a route to construct hybrid prototissues by incorporating i‐combisomes inside a fibrin network which serves as a surrogate for the extracellular matrix.

This report provides the first description of the fabrication, structure, and properties of i‐combisomes discovered from libraries of iCPs. The advanced level of biomimicry and the tunability of the i‐combisomes and their hybrids will undoubtedly serve as a platform to dissect and elucidate biological questions and design synthetic cells with non‐natural advanced functions.

## 2 Results and Discussion

### 2.1 Synthesis of Ionically‐Linked Comb Polymers

The iCPs were prepared in two steps: i) the polymerization of the backbone and ii) the complexation with DDP. The backbone was synthesized by single electron transfer‐living radical polymerization (SET‐LRP) of CBAA and DMAPAA in water at 0 °C via in situ reduction of Cu(II) to Cu(0).^[^ ^20^ ^]^ The generation of highly catalytically active Cu(0) nanoparticles afforded quantitative conversions as shown by kinetic studies (Figure S2, Supporting Information). The degree of polymerization and the molar ratio of DMAPAA was adjusted by varying the ratio of monomer to initiator and the ratio of CBAA to total monomer. The hydrophobic side chains were complexed by titration of the amine groups with DDP in methanol/chloroform to form combs. Details of the synthetic procedure, kinetics of polymerization, and molecular characterization can be found in the Supporting Information.

The modular synthesis of the iCPs enabled to design their molecular topology by varying the length of the hydrophilic backbone (*DP*) and the density of the hydrophobic grafts. The latter can be controlled by adjusting the content of DMAPAA (*N*) and the degree of substitution with DDP (*DS*). A library of iCPs was synthesized by systematically varying *DP*, *N*, and *DS* (Table S3, Supporting Information). We selected polymers with *DP* of 30, 85, 400, *N* between 15% to 70% and varied the *DS* between 50% and 100%. Hereafter, the iCPs will be named according to their molecular characteristic as follows: DP*_x_* N*_y_* DS*_z_* where *x* is the degree of polymerization, *y* is the molar percentage of DMAPAA, and *z* is the percentage of amine groups of DMAPAA complexed by DDP.

### 2.2 Self‐Assembly of iCPs

**Figure 2:**
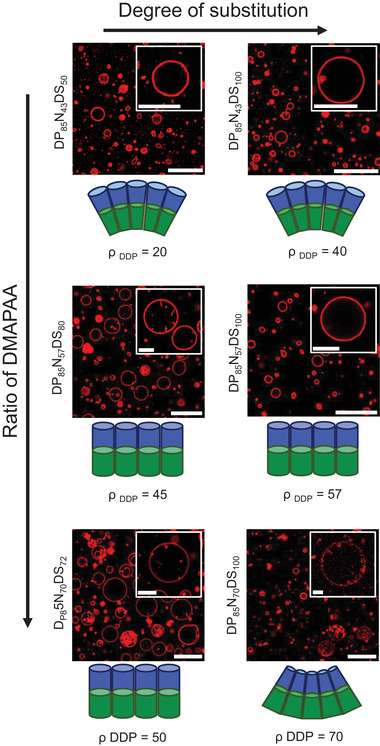
Self‐assembly of a library of iCPs with *DP*  =  85 in water studied by CLSM. The assembly was studied as a function of *ρ* _DDP_ which controls the packing parameter of an equivalent repeating unit depicted below the respective confocal images. *ρ* _DDP_ was adjusted by varying *N* and *DS*. The insets show an image of a representative i‐combisome. Scale bars are 30 µm for overview images and 10 µm for insets.

i‐Combisomes were assembled by the thin‐film rehydration or injection method in water. The former leads to the formation of giant unilamellar vesicles (2–20 µm) and the latter to small unilamellar vesicles (< 500 nm). Both methods resulted almost exclusively in unilamellar vesicles, and no onion vesicles were observed. Presumably, this is a consequence of the strong hydration of the carboxybetaine groups^[^ ^21^ ^]^ that prohibits the formation of stacked bilayers. The thin film rehydration method yielded i‐combisomes with a broad distribution of sizes (**Figure** **2**) typical for this method. On the other hand, the injection method afforded i‐combisomes with a very narrow dispersity in their size which could be controlled by the concentration of the iCPs (Figure S30, Supporting Information).

We used a library of iCPs with precise composition to dissect how their molecular structure controls the emergence of different self‐assembled structures in water. This work differs from previous works on polyelectrolyte‐surfactant complexes in which the complexation is carried out either in situ in water or to the preformed preferential lyotropic phase of the surfactant.^[^ ^19b,c,e^ ^]^ We studied the assembly as a function of the *DP* as well as the density of hydrophobic DDP tails across the backbone (*ρ* _DDP_ ≡ number of DDP in 100 monomers). The *ρ* _DDP_ was estimated by $\rho_{\mathrm{DDP}}=\frac{N\cdot DS}{100}$ which was tuned by varying *N* and *DS*. Figure 2 summarizes the different structures for *DP*  =  85 and varying *N* and *DS*.

No vesicle was observed when *ρ* _DDP_ was smaller than 20%. Cryogenic transmission electron microscopy (cryo‐TEM) and dynamic light scattering revealed the presence of micelles as the majoritarian assembly for *ρ* _DDP_  =  15 (DP_30_N_15_DS_100_, Figures S8 and S10, Supporting Information). Increasing the density of DDP in the range of 30–70% resulted in the formation of giant unilamellar vesicles. The same trend was observed for *DP*  =  30 and 400 despite their large disparity in molecular weights (Figures S8 and S9, Supporting Information). We hypothesize that we can predict the structure formation for iCPs using the packing parameter of an equivalent amphiphilic repeating unit analogously as for low molecular weight amphiphiles. This equivalent unit, schematically depicted in Figures S7 (Supporting Information), represents the smallest portion of the iCP that by translation can reproduce the whole polymer and controls the spontaneous curvature of the vesicle. This is in stark contrast to block copolymers in which the volume fraction of each block predicts which type of structure will be formed.^[^ ^9^, ^22^ ^]^

The most prominent morphology of giant i‐combisomes was spherical with rapid thermal fluctuations in their membrane. On the other hand, cryo‐TEM revealed that when i‐combisomes were prepared by injection method, a small fraction of the vesicles was faceted (Figure S12, Supporting Information). Faceted vesicles have been observed when an additional attractive force correlated the arrangement of synthetic lipids^[^ ^23^ ^]^ into a two‐dimensional nematic order with topological defects.^[^ ^24^ ^]^ Such linking of phospholipids into linear chains is the analogue of the backbone of the iCPs. We also observed the spontaneous formation of inward cylindrical nanotubes for those compositions with *DS* smaller than 100% (DP_85_N_43_DS_80_, DP_85_N_70_DS_72_, and DP_30_N_50_DS_80_). The formation of exclusively inward tubes indicates a slight imbalance in the area of the inner and outer leaflet and the concomitant generation of negative curvature.^[^ ^25^ ^]^ The incomplete complexation for *DS *< 100 may cause some backbone fragments to detach from the bilayer interface into the aqueous spaces. The driving force for detachment is larger in the outer leaflet than in the inner one given the larger water volume, generating an imbalance between the area of the leaflets that manifests as a nonlocal membrane bending as described by the “area difference elasticity model.”^[^ ^25^, ^26^ ^]^ Imbalances as low as 1% are sufficient to drive shape transformations in vesicles.^[^ ^27^ ^]^

**Figure 3:**
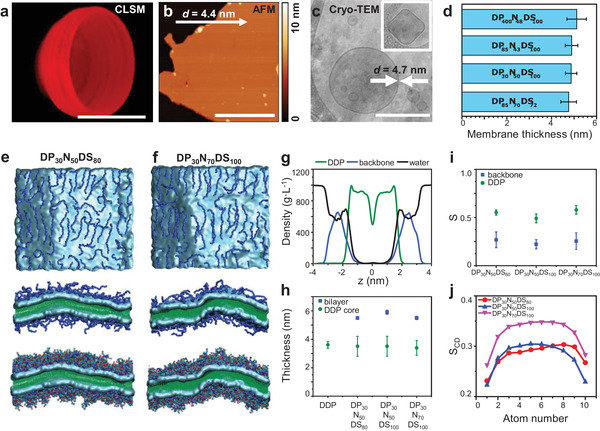
Molecular organization of iCPs at the i‐combisome membrane. a) 3D reconstruction of 70 confocal scans showing a bisected i‐combisome labeled with nile red (0.1 mol%). Scale bar: 10 µm. b) AFM height image of a deposited i‐combisome (DP_85_N_43_DS_100_) on mica at 55% RH. The bilayer height was analyzed along the arrow yielding a membrane thickness of 4.4 nm. Scale bar: 500 nm. c) Cryo‐TEM of i‐combisomes (DP_85_N_43_DS_100_) formed by the injection method with the respective membrane thickness. The inset shows an example of a faceted vesicle. Scale bar: 200 nm. d) The thickness of i‐combisome membranes was determined on multiple cryo‐TEM images and presented as the average of *n* = 15. e,f) Snapshots of simulated bilayers of DP_30_N_50_DS_80_ and DP_30_N_70_DS_100_. Top view images show the backbones (no explicit side groups) organized at the interface with water. The side views (cross‐sections along the normal to the membrane with and without visualization of the side groups) display the organization of the membrane with the backbone restricted to the interface with water. g) Density profile of DDP (green), polymer backbone (blue), and water (black) in i‐combisome DP_30_N_50_DS_80_. h) The simulated thickness of the DDP zone (green) and the total bilayer (blue). i) Orientational order parameter of the backbone (*S* ^backbone^, blue) and of DDP (*S* ^DDP^, green). j) Deuterium order parameter (*S*_CD_) for three different i‐combisomes.

Furthermore, by using cryo‐TEM and atomic force microscopy (AFM) we discovered that the thickness of the membrane was the same (≈5 nm) for all i‐combisomes regardless of the *DP* or molecular characteristics of the iCPs (**Figure** **3**b–d; and Figures S10 and S11, Supporting Information). On the other hand, in block copolymers, the formation of vesicles is usually restricted to polymers with lower molecular weights (10^3^–10^4^ g mol^−1^). The thickness of the bilayer scales with *DP* ^0.55^ rapidly reaching a thickness exceeding the ones of natural membranes.^[^ ^9^, ^22^ ^]^ But how could iCPs with drastically different *DP*s and molecular weights much higher than block copolymers lead to an invariant thickness? To tackle this question, we performed atomistic molecular dynamic simulations to study the molecular arrangements within the combisome membrane. The simulations revealed that the membrane consists of 3 zones: the core formed by a bilayer of DDP flanked by the backbones adsorbed on top of it (Figure 3e–g). The density profiles confirmed the markedly separated zones of the membranes. The DDP zone spans 2 nm around the midplane with almost no penetration of water or backbone. The backbone zones are concentrated at the interface with water, with ≈90% of the mass restricted to a 1.5 nm thick fully hydrated layer. This indicates that the backbones are confined to a 2D conformation. Examination of the cross‐sections and density profiles demonstrated that increasing the *ρ* _DDP_ was accompanied by the flattening of the backbone due to the higher number of attractive units. However, this variation resulted in only negligible changes in the total thickness of the membrane, well in line with the results of AFM and cryo‐TEM. The snapshots also show that the backbones assumed a rod‐like conformation organized in a nematic‐like fashion. Remarkably, this observation was confirmed for all samples studied. The ratio of the radii of gyration of the adsorbed backbones and the corresponding for a rod polymer ($R_{g}/R_{g}^{\mathrm{rod}}$) was close to one, (Figure S14, Supporting Information) confirming the rod conformation. This observation is in close agreement with previous theoretical work suggesting the stiffening of the backbone with the complexation of surfactants.^[^ ^19^, ^28^ ^]^ The stiffness prohibits the bending of the backbone and the formation of loops that could increase the thickness. Consequently, the thickness of the membrane is twice the height of the amphiphilic repeating unit and is decoupled from DP. We also estimated an orientational order parameter for these rods as $S^{\mathrm{backbone}}=\;\frac{\left\langle 3\cdot \cos^{2}\left(\psi \right)-1\right\rangle }{2}$ where *ψ* is the angle between the rods and the director. All i‐combisomes yielded *S* ^backbone^ > 0 (Figure 3i) demonstrating that the rods had a preferential orientation as shown in the snapshots which supports the observation of faceted vesicles (vide supra). Furthermore, we calculated the deuterium order parameter (*S*_CD_) and the orientation order parameter of the DDP (S^DDP^), which quantified the degree of order of the hydrophobic domains. All i‐combisomes displayed *S* ^DDP^> 0 and *S*_CD_ higher than for liposomes.

### 2.3 i‐Combisomes: A Biomimetic Liposome‐Polymersome Chimera

**Figure 4:**
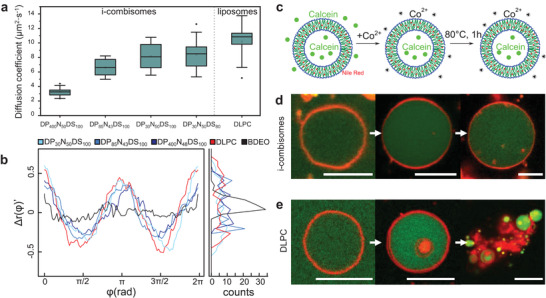
a) Boxplots for the diffusion coefficients of Rhod‐PE in supported bilayers of DP_85_N_43_DS_100_ and for the Rhod‐labeled backbone of DP_85_N_43_DS_100_. Boxes were generated from ten data points and contain the 25th to the 75th percentile of each data set. The line represents the median, while an open rectangle indicates the average. The whiskers show the standard deviation, while the outliers are displayed outside of the whiskers. b) Angular fluctuation of radii (Δ*r*(*φ*)’) after subtracting the first two harmonics of the cosine decomposition and distribution of the fluctuations (left). BDEO: polymersome from poly(BD_87_‐*b*‐EO_72_), DLPC: liposomes. c–e) Thermal stability assay: i‐combisomes (DP_85_N_43_DS_100_) and liposomes were formed in a calcein solution. The external calcein was quenched by the addition of a solution of Co^2+^. Subsequently, the temperature of the dispersion was risen to 80 °C for 1 h and cooled down before measurement. The i‐combisomes remained intact d), while liposomes underwent breakage and aggregation upon thermal treatment e). Scale bars: 10 µm.

To elucidate the effect of the molecular structure on the lateral mobility, we determined the diffusion coefficient (*D*) of a fluorescently labeled lipid (Rhod‐PE) within i‐combisome membranes by fluorescence recovery after photobleaching (FRAP) as depicted in **Figure** **4**a. All studied i‐combisomes showed *D* between 3 and 10 µm^2^ s^−1^, which are in the same range as for liposomes. Remarkably, the *D* of the rhodamine‐labeled backbone for DP_85_N_43_DS_100_ was also very close to the one observed for Rhod‐PE despite their large difference in size (Figure S15, Supporting Information). The lowest diffusion coefficient in the studied samples is between 10 and 1000 times higher than those for vesicles of block‐copolymers even though both systems are based on macromolecules.^[^ ^29^ ^]^ In i‐combisomes, the hydrophobic domains consist of the didodecyl alkyl tails in a liquid crystalline state above their melting temperature. Thus, the DDP tails and the fluorescently‐labeled lipid should be able to exchange positions with the neighbors as fast as in liposomes, i.e., millions of times per second.^[^ ^30^ ^]^ This led to *D* ≈ µm^2^ s^−1^ even for iCPs as large as 160 000 g mol^−1^ (DP_400_N_48_DS_100_). Such dynamics are not common for macromolecules of similar size. In polymersomes, the motion of the large hydrophobic blocks controls the *D*. For shorter hydrophobic blocks, the chains follow the Rouse model of motion, where *D* ≈ *DP* ^‐1^, while for large blocks the motion is dominated by reptation, where *D* ≈ *DP* ^‐2^.^[^ ^29a^ ^]^ However, in the i‐combisomes neither the hydrophobic tails nor the backbone have entanglements which could cause a reduction of the D with DP. Remarkably, we observed only a slight decrease of the diffusion coefficient with a 13‐fold increase in the *DP* for i‐combisomes with a similar density of DDP (Figure 4a). The molecular weight of this polymer is about 100 times higher than those of block copolymers widely used for vesicle formation.^[^ ^31^ ^]^ The *D* was insensitive with *DS* in the studied range. These findings are in agreement with the simulations showing no changes in DDP density inside the hydrophobic domains.

Another important property of cell membrane mimics is their flexibility. A large number of processes such as shape transformations, division, motion, endo‐, and exocytosis require the cellular membrane to be ultraflexible. In liposomes, the bending rigidity (*κ*_B_) measures the resistance to bend and is in the order of a few tens of the thermal energy (*κ*_B_ DOPC  =  19 *k*_B_ *T*)^[^ ^32^ ^]^ resulting in constantly fluctuating membranes. Similarly, the low phase transition temperature of the dodecane chains resulted in soft undulating membranes in all i‐combisomes (Video S1, Supporting Information). We further assessed the flexibility by analyzing the contours of the membranes of i‐combisomes. Figure 4b displays the angular distribution of radii (Δ*r*(*φ*)’) after subtraction of the size and shape contributions which allows to visualize the membrane fluctuations. All i‐combisomes displayed highly fluctuating membranes (Figure 4b; and Figures S16 and S17, Supporting Information) resembling those of soft liposomes such as DLPC, while hardly any fluctuations were observed for polymersomes of poly(BD_87_‐*b*‐EO_72_). The Δ*r*(*φ*)’ and the resulting histograms showed only slight differences in the fluctuation for i‐combisomes assembled from iCPs with a largely disparate degree of polymerization (30 < *DP* < 400). This is in contrast to polymersomes where the flexibility rapidly decreases with the increasing molecular weight. While in polymersomes bending requires the reorganization of the slow‐moving macromolecules,^[^ ^9b^ ^]^ in i‐combisomes it only requires the local reorganization of DDP which can freely ramble and exchange between neighboring chains, thus decoupling flexibility from the molecular weight.

The i‐combisomes displayed improved bench and thermal stability compared to liposomes. i‐Combisomes from DP_85_N_43_DS_100_ remained intact for at least 6 months stored at room temperature. The thermal stability was assessed by Confocal Laser Scanning Microscopy (CLSM) and Fluorescence Activated Cell Sorting (FACS) (Figure 4c–e; and Figure S18, Supporting Information). The addition of Co^2+^ to calcein‐filled i‐combisomes did not change the fluorescence intensity in the vesicle lumen, demonstrating that the membrane was impermeable to ions. Conversely, FACS showed a decrease in fluorescence for DLPC liposomes indicating a more permeable membrane. When a dispersion of i‐combisomes was heated to 80 °C the vesicles remained stable as shown by CLSM. The histogram of the fluorescent intensity confirmed that at least 80% were unchanged while the rest showed higher intensity, presumably due to fusion. On the other hand, liposomes underwent membrane breakage and aggregation. The breakage of the membrane allows Co^2+^ ions to quench calcein (Figure 4e; and Figure S18, Supporting Information).

Furthermore, we examined the pH and salt stability of i‐combisomes (Figures S28 and S29, Supporting Information). The i‐combisomes remained stable in HEPES (10 × 10^−3^ m) as well as in saline at physiological concentration (0.9 w%). However, increasing salt concentration to 1.8 w% led to changes in the morphology due to the shielding of the ionic interactions that connect the DDP to the backbone. On the other hand, the i‐combisomes displayed a remarkable pH stability. No changes in their morphology could be observed at pH 3 to 11. Only extreme pH below 2 led to morphological changes. Such a low pH may protonate the carboxylate groups changing the packing parameter.

### 2.4 Introducing Biological Functionality by Coassembly with Biomolecules

One possible route to endow synthetic cells with biological functionality is to integrate biological molecules or receptors into their membrane to allow recognition, signaling, or even reactions. However, the integration of such molecules demands sufficient biomimicry of the synthetic biomembranes. For example, the membrane must match the thickness of natural membranes to harbor functional lipids, peptides, or transmembrane proteins without hydrophobic mismatch.^[^ ^4c^ ^]^ Furthermore, the lateral mobility of these receptors has to be high to allow homogeneous mixing, formation of membrane complexes, as well as for multivalent interactions. Combining all these properties remains a challenge for most synthetic biomembranes precluding their use in synthetic biology. But can i‐combisomes fill this gap between liposomes and polymersomes?

#### 2.4.1 Lipid – i‐Combisome Hybrids

**Figure 5:**
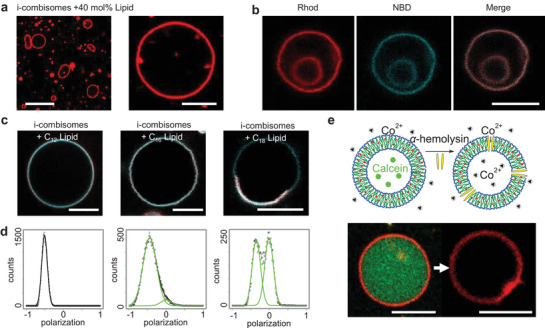
Coassembly with lipids a–d) and insertion of pore‐forming peptides e). a) Hybrid lipid—i‐combisomes containing 40 mol% Rhod‐PE (red). Scale bars: 20 and 5 µm. b) Hybrid lipid—i‐combisomes with rhod‐labeled iCP (red) and 16:0‐NBD‐PE lipid (cyan). Left: red channel (rhod), middle: cyan channel (NBD), right: merge. Scale bar: 5 µm. c,d) Coassembly with 20 mol% of lipids with different lengths of the hydrophobic tails: DLPC (C12), DPPC (C16), and DSPC (C18) labeled with Laurdan. c) Merged CLSM images of Laurdan emission detected at *λ*  =  415–445 nm (red) and *λ*  =  490–530 nm (cyan). Scale bars: 5 µm d) Distribution of the GP of Laurdan in lipid–i‐combisomes hybrid. e) Scheme depicting the insertion of *α*‐hemolysin and quenching of calcein fluorescence by Co^2+^ (top). i‐Combisomes labeled with nile red (membrane) and calcein (lumen) are contacted with Co^2+^. Spiking *α*‐hemolysin results in pore formation and the diffusion of Co^2+^ into the i‐combisome's lumen, quenching calcein. Scale bar: 5 µm. All i‐combisomes in this figure were formed from DP_85_N_43_DS_100_.

First, we assessed the ability to integrate lipids as constituents of the membrane. We formed hybrid vesicles by assembling DP_85_N_43_DS_100_ with the rhodamine‐labeled phospholipid Rhod‐PE (**Figure** **5**a; and Figure S19, Supporting Information) in a range of 0.1−40 mol%. CLSM images of all compositions revealed vesicles with red fluorescence across their membrane indicating that the lipid could homogeneously mix within the lipid—i‐combisome hybrid. Such high lipid ratios were shown to cause demixing, budding, and fission into daughter liposome and polymersome vesicles for similar compositions in PDMS‐*g*‐PEO.^[^ ^12a,g^ ^]^ To confirm that the observed vesicles were hybrids, we coassembled a 16:0‐NBD‐PE lipid (40 mol%) with DP_85_N_43_DS_100_ in which we labeled the backbone with rhodamine. Figure 5b demonstrates the colocalization of the green and red fluorescence stemming from the lipid and the iCP's backbone across the entire membrane. The facile mixing of these lipids in the i‐combisomes is a consequence of the excellent hydrophobic matching between PE lipid and the hydrophobic part of the iCPs.

Glycans form the third alphabet of life and have unsurpassed ability to encode biological functions. Hybrid glyco‐combisomes were prepared by the assembly of DP_85_N_43_DS_100_ with 16:0–18:1 DG glucose (20 mol%), which is a lipid containing glucose residues. Thin‐film rehydration afforded almost exclusively onion‐like glyco‐combisomes (Figure S21, Supporting Information). The preferential formation of onions suggests a strong association between bilayers driven by glycan–glycan interactions as observed for glycodendrimersomes.^[^ ^15c^ ^]^

#### 2.4.2 Raft‐Like Microdomains

We studied the generation of raft‐like microdomains by the coassembly with lipids by varying the length of their hydrophobic tails ranging from C12 to C18. On one hand, increasing the tail length increases the thermodynamic drive to form gel‐like bilayers (*L*_*β*_), while on the other hand it can impose a slight mismatch with the i‐combisome membrane and drive phase separation. To elucidate the degree of mixing we utilized Laurdan (1 mol%) as a fluorescent probe. Laurdan is a hydrophobic dye whose fluorescent spectrum is sensitive to the polarity of the surroundings and the exposure to water. We calculated the generalized polarization (GP) of Laurdan for each pixel of the hybrid membranes and determined the global GP distribution. The GP varies from +1 for no solvent effect and −1 for Laurdan in bulk water, which can be used to probe the ordering of the membrane.^[^ ^33^ ^]^

CLSM images of pure i‐combisomes labeled with Laurdan displayed homogeneous GP across the entire membrane with a monomodal GP distribution centered at −0.46 (Figure S20, Supporting Information). This confirms that Laurdan could be molecularly mixed in the membrane of the i‐combisomes. The coassembly with 20% of DLPC (C12) or DPPC (C16) also resulted in a homogeneous GP. However, while the GP distribution of DLPC was monomodal (centered at −0.50), the one with DPPC could be deconvoluted into two contributions: a disordered one at −0.46 and a more ordered one at +0.10 (Figure 5c,d). This indicates that, while DLPC completely mixed, the longer hydrophobic domains in DPPC resulted in ordered nanodomains homogeneously distributed across a more disordered phase. On the other hand, the i‐combisomes containing DSPC (C18) displayed microscopic patches with pronounced differences in the GP. The global distribution of the GP was bimodal with one peak roughly corresponding to the one of the pure i‐combisome and a second one displaying a GP of +0.1. The latter stems from the generation of C18‐rich L_*β*_ microdomains surrounded by an iCP‐rich *L*_*α*_ continuous domain. It is counterintuitive that only DSPC generated microdomains and not DPPC, despite both having melting temperatures well above room temperature. This suggests that only the longer C18 chains of the DSPC could generate sufficient hydrophobic mismatch to drive the phase separation into the more ordered and thermodynamically more stable *L*_*β*_. This demonstrates that microdomains can be readily formed in the i‐combisomes by a judicious choice of the molecular structure of the lipids.

#### 2.4.3 Pore Formation at the i‐Combisome Membrane

The addition of *α*‐hemolysin to calcein‐filled i‐combisomes resulted in the rapid quenching of the green fluorescence by Co^2+^ indicating the formation of a functional pore (Figure 5e). The ability of the i‐combisome to accommodate the insertion of this peptide and its assembly into a homoheptameric pore unit requires matching of the hydrophobic domains, lateral mobility, and mixing with the iCPs, highlighting the biomimetic nature of the i‐combisome membranes.

### 2.5 Fusion with Liposomes

Membrane fusion is a fundamental process of living organisms. Vesicles act as supramolecular shuttles carrying functional macromolecules that they deliver to other cells and organelles by fusion.^[^ ^3a^ ^]^ For example, the fusion of vesicles is central in protein labeling and sorting, in cell‐to‐cell communications, synaptic transmission, and viral infection, to mention a few.^[^ ^34^ ^]^ Moreover, vesicle fusion is arguably the easiest way to introduce new membrane material and receptors to synthetic cells.^[^ ^12^, ^35^ ^]^

**Figure 6:**
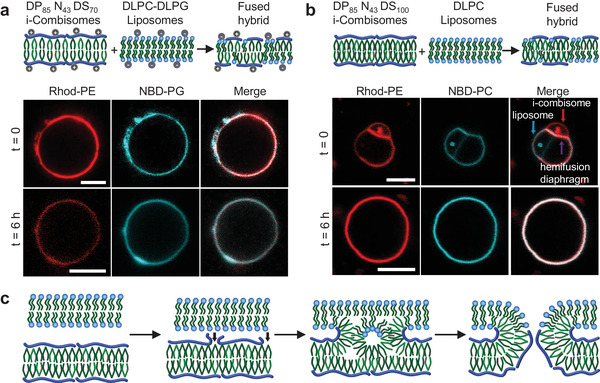
a) Electrostatically‐driven fusion of anionic liposomes (DLPC‐DLPG, 8:2) and cationic i‐combisomes (DP_85_N_43_DS_70_). The liposomes and i‐combisomes are labeled with NBD‐PG and Rhod‐PE, respectively. CLSM of representative vesicles immediately after mixing and after 6 h. Left: red channel, middle: cyan channel, right: merge. b) Fusion of i‐combisomes (DP_85_N_43_DS_100_) and liposomes (DLPC) assembled from electroneutral components labeled with Rhod‐PE and NBD‐PC. CLSM images of representative vesicles immediately after mixing and after 6 h. At time zero it was possible to observe the hemifusion diaphragm. Left: red channel, middle: green channel, right: merge. Scale bars: 5 µm. c) Scheme of the model of fusion between lipids (top bilayer) and i‐combisome (bottom bilayer) highlighting the steps.

First, we monitored the interactions of positively charged i‐combisomes (red, Rhod‐PE) with negatively charged liposomes (cyan, NBD‐PG) using CLSM (**Figure** **6**a). The charged i‐combisomes were assembled from cationic DP_85_N_43_DS_70_, which has 10 cationic residues per polymer chain, while the negatively charged liposomes were formed from a lipid mixture of neutral DLPC and anionic DLPG (8:2). In control experiments, the two vesicle types were imaged separately to ensure that the rhod and NBD signals did not overlap (Figure S23, Supporting Information). Immediately after mixing and without any additional shaking we could only observe i‐combisomes (red) with cyan patches and no more free liposomes. This indicates that all liposomes fused with the i‐combisomes. After 60 min, the patches became smaller and completely disappeared after 6 h, resulting in the colocalization of cyan and red fluorescence across the whole surface of the fused vesicles. This demonstrates the homogeneous mixing at the microscopic level. Furthermore, a small fraction of the liposomes was oligovesicular. After mixing with i‐combisomes we observed red fluorescence only in the external membrane. This supports the fusion process over an engulfment mechanism (Figure S24c, Supporting Information).

Then, we examined whether the fusion was possible for i‐combisomes and liposomes assembled from electroneutral DP_85_N_43_DS_100_ (red, Rhod‐PE) and DLPC (cyan, NBD‐PC). The lack of strong electrostatic drive resulted in a slower process compared to the charged ones and allowed observing the intermediate stage of hemifusion (Figure 6b; and Figure S24a,b, Supporting Information). After a few minutes, the smaller i‐combisome adhered to the larger liposome resulting in a hemifusion diaphragm, characterized by strong colocalization of the cyan and red fluorescence. Some red dye was also transported from the i‐combisome to the liposome. However, we did not observe a transport of the cyan dye from the liposome to the i‐combisome during this observation time. The hemifusion evolved to complete fusion after 6 h. Lipids and iCPs were homogeneously mixed in the resulting hybrid vesicle as evidenced by the colocalization of dyes across the whole membrane.

Previous attempts to fuse vesicles always required the presence of fusogenic membrane components (SNARE, peptides, DNA)^[^ ^12^, ^35^, ^36^ ^]^ or an addition of salts or polymers and energy input to overcome the barriers to fusion,^[^ ^12^, ^37^ ^]^ with only scarce reports for polymersomes/liposomes fusion. Moreover, in the case of PDMS‐*g*‐PEG, the mechanically‐induced fusion with lipids was immediately followed by fission into daughter vesicles of lipids and polymers.^[^ ^12b^ ^]^ Thus, what is behind the capacity of i‐combisomes to readily fuse with liposomes while remaining stable?

Fusion requires that the membranes approach, adhere, and form an unstable nonbilayer intermediate that evolves into a fusion pore.^[^ ^38^ ^]^ The approach and adhesion require attractive interactions surpassing the repulsion between hydrophilic groups of the membrane.^[^ ^39^ ^]^ This can be driven by the local release of backbone fragments and by creating transient hydrophobic point defects by exposing DDP to the water phase (Figure 6c). This would result in a focal adhesion of the two apposed membranes. Compared to phospholipids this is facilitated by the intrinsic flexibility of the i‐combisomes and the increase in configurational entropy when the backbone is partly released and no longer solely confined to the 2D interface. Afterward, the theory describes the formation of an inverted micellar intermediate (IMI) followed by interlamellar attachment (fusion pore).^[^ ^40^ ^]^ The formation of these intermediates requires amphiphiles with either negative spontaneous curvature or the capability to reorganize. In cell membranes, this is enabled by nonvesicle forming molecules.^[^ ^41^ ^]^ Presumably, in i‐combisomes, the non‐directional nature of the ionic bond between hydrophilic and hydrophobic parts allows the local reorganization of the iCPs at the contact point by increasing the density of DDP (Figure 6c). This transient topological reorganization would result in negative curvature that reduces the kinetic barrier for IMI formation by the favorable increase in entropy of the backbone and the good packing that can be achieved by the DDP in the IMI. This should endow the i‐combisomes with a more favorable pathway to undergo fusion with other vesicles.

### 2.6 Hybrid i‐Combisome Protocells

**Figure 7:**
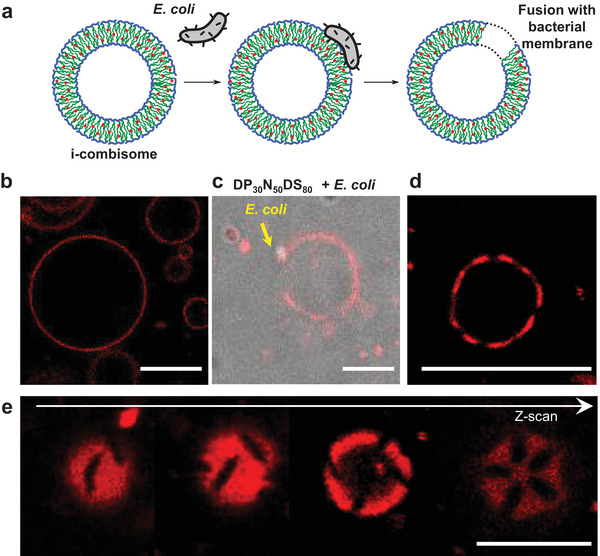
Fusion of i‐combisomes with living *E. coli*. a) Scheme demonstrating the adhesion and fusion process. b) i‐Combisomes before contact with bacteria. c) Adhesion of *E. coli* after 2 min. d) Formation of patches after fusion. e) Z‐scan of a hybrid vesicle showing elliptical patches. Scale bar: 5 µm.

Here, we examined the fusion of the i‐combisomes with living matter (**Figure** **7**). Fusion with living cells can be considered as a route to "hijack" the extremely complex macromolecular periphery of cells and capture the molecules of their cytoplasm in the lumen of the fused hybrid vesicles. Figure 7 shows the interaction of living *E. coli* with nile‐red‐labeled i‐combisomes. Almost immediately after mixing multiple bacteria adhered to the surface of the i‐combisomes (Figure 7c). A few minutes afterward the adhered bacteria merged with the i‐combisomes resulting in the formation of dark elliptical patches across the surface of the membrane (Figure 7d,e). These patches are the result of the fusion of the i‐combisome membrane with the outer‐membrane layer (OML) of *E. coli*. The OML layer is an asymmetric membrane in which the external leaflet is rich in rigid liposaccharides that drive the segregation from the iCPs and form phase‐separated stable patches.

The cell surface makeup of natural cells governs cell–cell interactions. The fabrication of hybrids of i‐combisomes and bacteria by fusion enabled to readily combine the chemical and biological surface design in a single protocell. This methodology allows for capturing the active receptors of bacteria membrane without the need for any purification step. Moreover, we developed a methodology to fabricate a basic prototissue based on i‐combisomes embedded in a surrogate of an extracellular matrix (Figure S27, Supporting Information). The vesicles were intimately connected to the matrix in a focal‐adhesion‐like fashion.

## 3 Conclusions

We introduced a concept for cell membrane mimics based on the self‐assembly of a new class of amphiphilic comb polymers, iCPs. These combs have a hydrophilic zwitterionic backbone to which hydrophobic tails are electrostatically linked. Using a combination of optical, electron, and force microscopies, we investigated the self‐assembly of a library of iCPs in water. We discovered the prominent formation of vesicular structures with membrane thicknesses independent of the *DP* and *DS* of the iCPs which closely mimic those of their low molecular weight counterparts–phospholipids. Atomistic molecular dynamic simulations allowed to elucidate the molecular organization within the i‐combisome membranes. The *L*_*α*_ structure of DDP within the core of the membrane forces the backbone into a rod conformation confined to two dimensions with nematic‐like ordering. Contrary to the belief that molecular flexibility is a necessary condition to form flexible vesicles, we demonstrated that highly stiff rods assembled into stable i‐combisomes with the flexibility and membrane dynamics on par with ultraflexible DLPC liposomes.

The accurate matching of the thickness and lateral mobility of cell membranes as well as the ability to locally reconfigure the molecular topology of its constituent endowed the i‐combisomes with an unparalleled ability to seamlessly integrate functional components of natural membranes. This was demonstrated by the coassembly with fully mixable and structure‐directing (raft and onions) (glyco)lipids as well as by the incorporation of transmembrane pores and the "hijacking" of the cell periphery of living *E. coli* by fusion. We envision that the high level of biomimicry, the tunability of the chemical and biological makeup of the membrane and the ability to fuse with living matter will result in synthetic cells with augmented functions that can be used to probe complex biological questions and open up new concepts in biomedicine, and will result in a platform for drug delivery vehicles.

## Statistical Analysis

All of the reported experiments were performed at least twice to confirm the reproducibility of the results. All directly measured data are presented without preprocessing unless stated otherwise. The statistical data shown in Figure 3d were obtained as the average of 15 thicknesses. Data were expressed as mean ± standard deviation. The boxplots in Figure 4a were generated from 10 data points containing 25–75% of the data set. In Figures 4b and 5c,d, the displayed data were obtained from one representative vesicle each. The data were processed according to the description in the respective Supporting Information Section. Statistical analysis and data fitting were performed in OriginPro2018, Python, and R.

## Conflict of Interest

The authors declare no conflict of interest.

## Supporting information

Supporting Information

Supplemental Video 1

## Data Availability

The data that support the findings of this study are available from the corresponding author upon reasonable request.
