# Supplementary material for: Ionic Combisomes: A New Class of Biomimetic Vesicles to Fuse with Life
Source: Adv Sci (Weinh). 2022 Apr 7;9(17):2200617. doi: 10.1002/advs.202200617 (PMC9189634; doi:10.1002/advs.202200617)
Supplement: Supplementary file 1 — Supporting Information [file ADVS-9-2200617-s001.pdf]

ADVANCED SCIENCE
Open Access

Supporting Information

for *Adv. Sci.*, DOI 10.1002/advs.202200617

Ionic Combisomes: A New Class of Biomimetic Vesicles to Fuse with Life

*Anna M. Wagner, Jonas Quandt, Dominik Söder, Manuela Garay-Sarmiento, Anton Joseph, Vladislav S. Petrovskii, Lena Witzdam, Thomas Hammoor, Philipp Steitz, Tamás Haraszti, Igor I. Potemkin, Nina Yu. Kostina, Andreas Herrmann and Cesar Rodriguez-Emmenegger**

Supporting Information

Ionic combisomes: A new class of biomimetic vesicles to fuse with life

Anna M. Wagner, [†] *Jonas Quandt,* [†] *Dominik Söder, Manuela Garay-Sarmiento, Anton Joseph, Vladislav S. Petrovskii, Lena Witzdam, Thomas Hammoor, Philipp Steitz, Tamás Haraszti, Igor I. Potemkin, Nina Yu. Kostina, Andreas Herrmann and Cesar Rodriguez-Emmenegger**

[†]These authors equally contributed to this work

A. M. Wagner, J. Quandt, D. Söder, M. Garay-Sarmiento, A. Joseph, L. Witzdam, T. Hammoor, P. Steitz, T. Haraszti, N. Yu. Kostina, I. I. Potemkin, A. Herrmann, C. Rodriguez-Emmenegger
DWI – Leibniz Institute for Interactive Materials, Forckenbeckstraße 50, 52074 Aachen, Germany
E-mail: rodriguez@dwi.rwth-aachen.de

A. M. Wagner, J. Quandt, D. Söder, A. Joseph, L. Witzdam, N. Yu. Kostina, A. Herrmann
Institute of Technical and Macromolecular Chemistry, RWTH Aachen University, Worringerweg 2, 52074 Aachen, Germany

C. Rodriguez-Emmenegger
Institute for Bioengineering of Catalonia (IBEC), Carrer de Baldiri Reixac, 10, 12, 08028 Barcelona, Spain.

C. Rodriguez-Emmenegger
Institució Catalana de Recerca i Estudis Avançats (ICREA), Passeig Lluís Companys 23, 08010 Barcelona, Spain

M. Garay-Sarmiento
Chair of Biotechnology, RWTH Aachen University, Worringerweg 3, 52074 Aachen, Germany

V. S. Petrovskii, I. I. Potemkin
Physics Department, Lomonosov Moscow State University, Leninskie Gory 1-2, 119991 Moscow, Russian Federation

I. I. Potemkin
National Research South Ural State University, Chelyabinsk 454080, Russian Federation

1. Materials & Methods

Materials:

All materials were as received or otherwise stated.

Table S1. Materials and their source

Solketal	Sigma-Aldrich
Anhydrous THF	Thermo Scientific
2-Bromoisobutylryl-bromide	Sigma-Aldrich
(2,2-dimethyl-1,3-dioxolan-4-yl)methyl 2-bromo-2-methylpropanoate	Sigma-Aldrich
Diethylether	Merck
Glacial acetic acid	Merck
Hydrochloric acid	VWR
Sodium hydrogen carbonate	VWR BDH Chemicals
Magnesium sulfate	VWR BDH Chemicals
Anisole	Sigma-Aldrich
N-[3-(dimethylamino)propyl] acrylamide	TCI
Toluene	VWR BDH Chemicals
β-Propiolactone	Sigma-Aldrich
n-Hexane	Fischer Chemicals
Rhodamine-123	Sigma-Aldrich
EDC	Sigma-Aldrich
Copper (II) Bromide	Sigma-Aldrich
Sodium Borohydride	Sigma-Aldrich
Me_6TREN	Sigma-Aldrich
Anhydrous methanol	Acros Organics
Nile Red	Sigma-Aldrich
Calcein	Sigma-Aldrich
Human fibrinogen	Sigma-Aldrich
Human thrombin	Sigma-Aldrich
Laurdan	Sigma-Aldrich
Tris-buffer (pH = 7.4)	Sigma-Aldrich
α-Hemolysin	Sigma Aldrich

HEPES	PenReac AppliChem
Anhydrous chloroform	Acros Organics
Didodecyl hydrogen phosphate	abcr
Bodipy FL	Sigma Aldrich
LB medium ingredients	AppliChem GmbH
Kanamycin	AppliChem GmbH
Cobalt chloride hexahydrate	Sigma Aldrich
E. coli BL21 (DE3) Gold	Agilent Technologies Inc.
FITC-fibrinogen	Zedira
Ethyl acetate	Acros organics
Atto-620 DNA	Biomers
Poly (butadiene-block-ethylene oxide)	Polymer Source, Inc.
12:0 PC (DLPC) 1,2-dilauroyl-sn-glycero-3-phosphocholine	Avanti lipids
12:0 PG (DLPG) 1,2-dilauroyl-sn-glycero-3-phospho-(1'-rac-glycerol) (sodium salt)	Avanti lipids
16:0 PC (DPPC) 1,2-dipalmitoyl-sn-glycero-3-phosphocholine	Avanti lipids
18:0 PC (DSPC) 1,2-distearoyl-sn-glycero-3-phosphocholine	Avanti lipids
16:0 NBD PE 1,2-dipalmitoyl-sn-glycero-3-phosphoethanolamine-N-(7-nitro-2-1,3-benzoxadiazol-4-yl) (ammonium salt)	Avanti lipids
14:0-06:0 NBD PG 1-myristoyl-2-{6-[(7-nitro-2-1,3-benzoxadiazol-4-yl)amino]hexanoyl}-sn-glycero-3-[phospho-rac-(1-glycerol)] (ammonium salt)	Avanti lipids
14:0-06:0 NBD PC 1-myristoyl-2-{6-[(7-nitro-2-1,3-benzoxadiazol-4-yl)amino]hexanoyl}-sn-glycero-3-phosphocholine	Avanti lipids

Synthesis and molecular characterization

Synthesis of 2,3-dihydroxypropyl 2-bromo-2-methylpropanoate (Initiator): Solketal (1, 4.7 mL, 0.0378 mol, 1 eq.), freshly distilled Et_3N (11 mL, 0.0789 mol, 2.1 eq.) and dry THF (33 mL) were transferred into a dry Schlenk flask in countercurrent of Argon. 2-Bromoisobutylryl-bromide (5.13 mL, 0.0415 mol, 1.1 eq.) was dissolved in dry THF (14 mL) and added dropwise to the solketal solution at 0°C. The ice bath was removed and the solution was stirred overnight. The solvent was removed by reduced pressure and the residue was slurried with Et_2O and filtrated. The filtrate was washed with 10 % HCl. The organic phase was washed with brine and saturated $NaHCO_3$. The organic phase was dried over MgSO4 and the solvent removed to yield (2,2-dimethyl-1,3-dioxolan-4-yl)methyl 2-bromo-2-methylpropanoate as an orange oil (8.48 g, 0.0302 mol, 80 %). The oil was dissolved in a mixture of deionized water (80 mL) and glacial acetic acid (30 mL) and anisole (0.05 mL) were added. The mixture was stirred for one hour at 80 °C. The reaction solution was extracted by ethyl acetate and the aqueous phase was saturated with $NaHCO_3$, filtered and extracted three times with ethyl acetate. The organic phases were mixed and the solvent was removed under reduced pressure. A yellow oil was obtained which crystallized overnight. For recrystallization, 70 mL Toluene was added and heated up to 60 °C for one hour. After recrystallization overnight white crystals (3, 4.4181 g, 0.0183 mol, 61 %) were obtained. ^{1}H-NMR (300 MHz, DMSO): δ = 4.96 ppm (d, J = 5.3 Hz, 1H), 4.67 ppm (t, J = 5.7 Hz, 1 H), 4.07 ppm (m, 2 H), 3.68 ppm (sextet, J= 5.2, 1 H),3.37 ppm (td, J = 5.7, 1.6 Hz, 2H), 1.90 ppm (s, 6 H).

Synthesis of 3-((3-acrylamidopropyl) dimethylammonio)propanoate (CBAA): CBAA was prepared by a previously reported procedure.[1] In short *N*-[3-(dimethylamino)propyl] acrylamide (DMAPAA 49,99 g, 320 mmol, 1 eq.) was dissolved in 200 mL of dry THF and cooled to 0 °C. β-propiolactone (28,65 g, 397,56 mmol, 1,25) was dissolved in 90 mL of dry THF and added dropwise under inert atmosphere for 2 h. The reaction was allowed to warm up to room temperature and proceeded for 24 h. The white precipitate was filtered-off and subsequently washed with dry THF and ether. The product was dried under high vaccum for 24 h. ^{1}H NMR (400 MHz, Deuterium Oxide): δ 6.31 – 6.12 (m, 2H), 5.76 (dd, J = 9.6, 2.0 Hz, 1H), 3.54 (dd, J = 8.5, 7.1 Hz, 2H), 3.40 – 3.28 (m, 4H), 3.05 (s, 6H), 2.68 – 2.59 (m, 2H), 2.09 – 1.97 (m, 2H). ^{13}C NMR (101 MHz, Deuterium Oxide): δ 176.38 (d, J = 1.7 Hz), 168.65, 129.74, 127.59, 62.40 – 61.59 (m), 61.42 – 61.03 (m), 51.39 – 49.93 (m), 37.39 – 34.21 (m), 30.69, 22.18.

Synthesis of Rhodamine-123 labeled CBAA-co-DMAPAA copolymer: Copolymer $DP_{85}N_{43}$ (8.46 mg, 0.025 mmol CBAA, 1.0 eq) and $DP_{30}N_{50}$ (9.61 mg, 0.025 mmol CBAA, 1.0 eq.) were dissolved in 1 mL of water each. Rhodamine-123 (9.52 mg, 0.025 mmol, 1.0 eq) was dissolved in aq. HCl (8.0 mL, pH = 4-5) and added to the polymer solution. To the solution EDC (7.06 mg, 0.050 mmol, 2.0 eq.) was added under vigorous stirring. The reaction was stirred over night at room temperature. Afterwards the reaction mixture was dialyzed against aqueous HCl (pH = 5) for 24 hours. The labeled backbone was obtained as violet solid after freeze drying and stored under inert gas until further use.

Synthesis of the CBAA-co-DMAPAA copolymer: $CuBr_2$ (16.6 mg, 0.074 mmol, 0.8 eq.) and $NaBH_4$ (1.4 mg, 0.037 mmol, 0.4 eq.) were added into a Schlenk flask equipped with a magnetic stirring bar. In a vial, Milli-Q-water (1.00 mL) and Me_6TREN (29.8 µL, 0.111 mmol, 1.2 eq.) were mixed. Both vessels were sealed and degassed by sparging with argon for 30 minutes. The aqueous solution was transferred with a gas-tight syringe under inert atmosphere to the solids and the mixture was stirred for 30 minutes at 0 °C to allow complete formation of Cu(0)-particles. Simultaneously, *N*-[3-(dimethylamino)propyl]acrylamide (DMAPAA) was passed over an aluminum oxide column to remove the inhibitors and then dissolved in water (2.48 mL). In case of polymers DP = 30 and DP = 400, DMAPAA was protonated with an equivalent amount of acetic acid. (3-Acryloylamino-propyl)-(2-carboxy-ethyl)-dimethyl-ammonium (CBAA) and the initiator (22.4 mg, 0.093 mmol, 1.0 eq.) were added, degassed with argon for 30 minutes at 0 °C and transferred to the Cu(0)-particles under inert gas atmosphere. The polymerization was stirred at 0 °C for 30 minutes. The polymerization was terminated by exposure to air and freezing with liquid nitrogen. The Cu(0)-particles were removed by centrifugation and the supernatant is dialyzed for 2 days against acidified water (3x water change/day, MWCO = 1 kDa). Freeze-drying yielded the purified copolymer as colorless solid.

Synthesis of ionically-linked comb polymers (iCPs): Copolymers were complexed with DDP via an acid-base reaction to form iCPs. An organic solution of DDP in $CHCl_3$ (c = 40 $mg{\cdot}mL^{-1}$) was added dropwise to a stirring solution of copolymer in MeOH (c = 10 $mg{\cdot}mL^{-1}$) at room temperature. The amount of DDP was adjusted to obtain the desired DS. After 2 hours stirring the solvent was evaporated and the iCPs were dissolved in MeOH at 10 $mg{\cdot}mL^{-1}$ (MeOH/$CHCl_3$ 50/50 for compositions not soluble in pure MeOH) and stored at 4 °C.

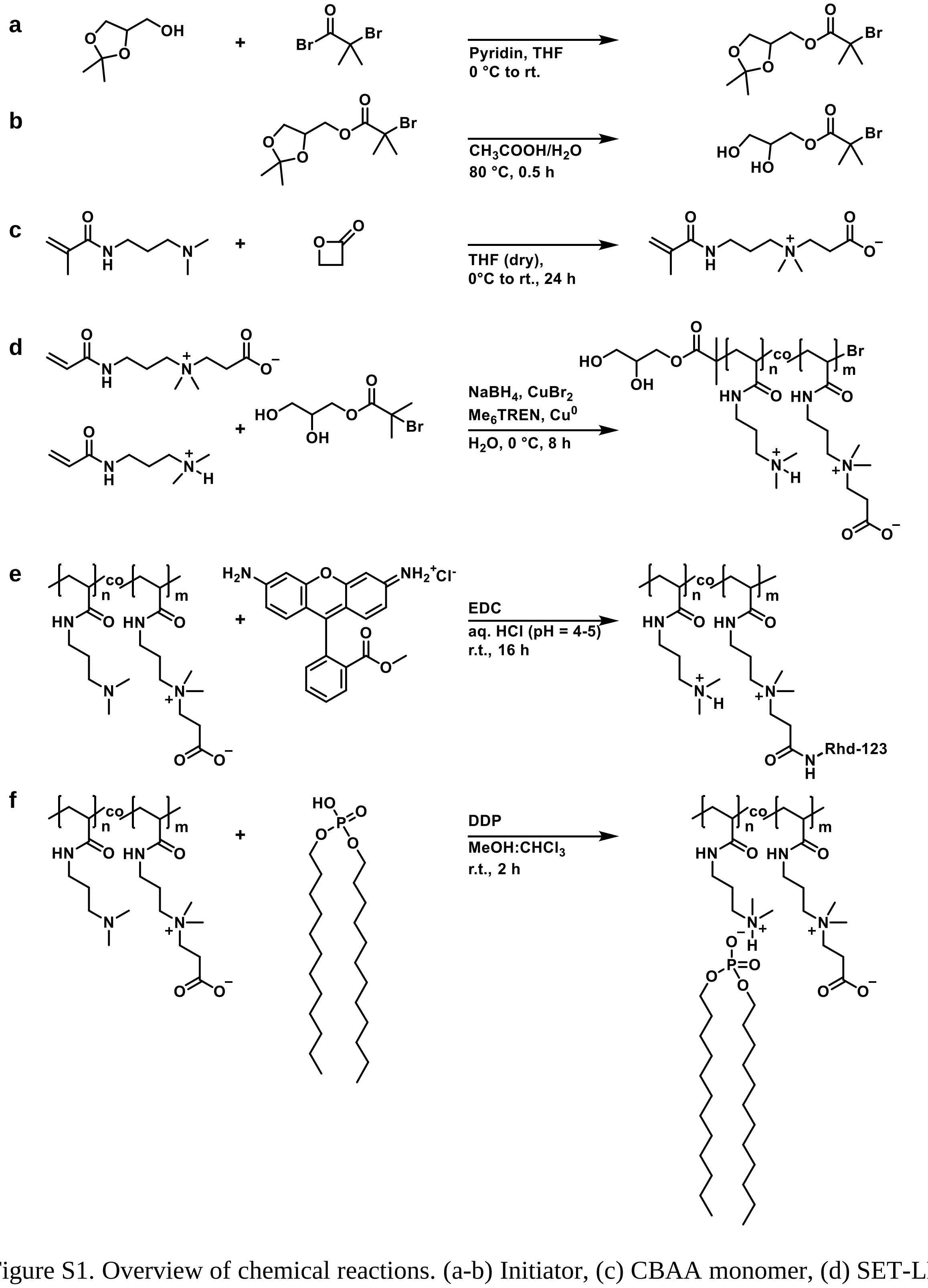

Figure S1. Overview of chemical reactions. (a-b) Initiator, (c) CBAA monomer, (d) SET-LRP of CBAA-*co*-DMAPAA, (e) Rhodamine-labeling, (f) Complexation with DDP

Nuclear magnetic resonance spectroscopy (NMR): All synthesized compounds were characterized by recording their NMR spectra at room temperature using a Bruker AvanceIII 400. Spectra analysis was performed with MestReNova V.12.0.1. The chemical shifts are reported in δ units relative to the solvent residual peak (CD_3OD: δ_H = 3.31 ppm, δ_C = 49.0 ppm, $CDCl_3$: δ_H = 7.26 ppm, δ_C = 77.16 ppm, D_2O: δ_H = 4.79 ppm)[2]. After referencing of the respective ^{1}H-NMR spectrum the MestReNova software was used for ^{31}P-NMR spectra absolute referencing. The following abbreviations were used throughout: s = singlet, d = doublet, t = triplet, q = quartet, dd = doublet of doublet, etc., m = multiplet. Coupling constants (*J*) are given in Hz.

Size exclusion chromatography (SEC): The weight-average molecular weight (M_w^{SEC}), the number-average molecular weight (M_n^{SEC}), and the polydispersity indices (M_n/M_w; PDI) were determined by SEC using water with NaCl (0.1 M), TFA (0,3 Vol%) and NaN_3 (0,01w%) as eluent in an Agilent-1200 system, equipped with a degasser, isocratic pump, manual injection valve, refractive index detector. Calibration was achieved using poly(ethylene glycol) standards. Ethylene glycol (Fluka) was used as internal standard. Samples of 25 µL were injected at room temperature with a concentration of 5 mg·mL^{-1}. A series of Nomvema Max Lux columns (PSS) was used for the separation. Data analysis was done with PSS WinGPC UniChrom software (Version 8.1.1).

Vesicle preparation.

Thin film rehydration method: Bilayer vesicles (i-combisomes) were self-assembled from iCPs *via* thin film rehydration. An organic solution of amphiphiles in MeOH (100 µL, *c* = 10 mg·mL^{-1}) was deposited on a roughened Teflon plate and dried at ambient conditions (30 min). Afterwards the samples were dried for 2 h *in vacuo*. Milli-Q water was added onto the Teflon plate to rehydrate the dried amphiphile film (c = 1 mg·mL^{-1}) at 60 °C for overnight.

Injection method: i-combisomes were prepared by injection for the characterization by cryo-TEM, DLS, and FACS. A MeOH solution of amphiphilic combs (100 µL, *c* = 10 mg·mL^{-1}) was injected into water (c = 0.5 mg·mL^{-1}) and vortexed for 20 s. The vial was left open at ambient conditions for 24 h to evaporate the remaining MeOH prior to use.

Vesicle characterization

Confocal Laser Scanning Microscopy (CLSM): CLSM was used to study the morphology, size and interactions of large i-combisomes prepared by thin-film hydration. Images and videos of the vesicle populations were recorded on a Leica TCS SP8 confocal microscope (Wetzlar, Germany) with a 63x/1.40 glycerol-immersion objective and a HyD detector. The measurements were performed at a constant temperature of 22 °C. An iCP dispersion (20 µL, 1 mg·mL-1) was deposited between two high-precision microscope cover glasses (170 ± 5 µm) for the sample preparation. The cover glasses were sealed with Secure-Seal™ spacers (13 mm diameter, 0.12 mm deep). All images were corrected in contrast and brightness and cropped using the Fiji-ImageJ software.

Atomic force microscopy (*AFM*)*:* A diluted dispersion of i-combisomes prepared by thin-film hydration in water (c_{diluted} = 0.5 mg·mL^{-1}) was drop casted on freshly peeled Mica. The film was dried in a humidity-controlled chamber from PicoPlus (Molecular Imaging, Agilent Technologies, USA) for 12 h at 25 °C and 55% relative humidity. Images were recorded in the chamber under the same conditions (25 C, 55% relative humidity) on a Agilent Series 5500 (Agilent Technologies, USA) as topological scans in tapping mode in air, using OTESPA-R3 silicon probes (Bruker, France) with a nominal spring constant of 26 N·m^{-1} and a tip radius of 7 nm. The images were analyzed using the Gwyddion software[3].

Cryogenic transmission electron microscopy (Cryo-TEM): Cryo-TEM was used to study the morphologies of vesicles prepared by injection method. The samples were measured at -168 °C with an applied electron beam acceleration voltage of 120 kV on a Zeiss LibraTM 120 transmission electron microscope (Oberkochen, Germany) in cryogenic mode. The samples were prepared using a FEI Vitrobot (Model Mark IV) plunge freezing station by deposition of an aqueous i-combisome dispersion (5 µL, 1 mg·mL^{-1}) on plasma-treated lacey grids. After blotting and shock freezing in liquid ethane, the samples were fixed on a Model 910 cryo transfer specimen holder from Gatan (Pleasanton, California). Images were taken using an in-column Omega energy filter with a CCD detector.

Dynamic light scattering (DLS): DLS was used to determine the hydrodynamic diameter (D_h) of small objects resulting from the assembly of iCPs. Measurements were performed on a Zetasizer Ultra from Malvern Instruments using a fixed scattering angle of θ = 90°, a laser beam operating at λ = 632.8 nm and using DTS0012 disposable cuvettes. Before each

measurement, samples were equilibrated at 25 °C for 90 s. Multiple data acquisitions (> 3) were performed for each sample. Data processing was performed by the software supplied with the Instrument (ZS XPLORER 1.2.0.91).

Molecular dynamics simulation: Geometrical models cannot provide an accurate representation of the self-assembly of iCPs due to their complexity and size. To gain insight in the i-combisome structure we performed atomistic molecular dynamic simulations. Molecular dynamics were carried out using the Gromacs 2019 package with a constant temperature of 300 K.[4] The NPT ensemble at the pressure 1 atm was used to obtain a local thermodynamic equilibrium, and the further simulation was continued in the NVT ensemble. In both ensembles, the temperature is coupled by a velocity–rescale thermostat,[5] and pressure coupling is set by Berendsen barostat. Periodic boundary conditions were applied in all directions. The motion equations were integrated with a time step of 2 fs. The cut-off length of Lennard-Jones potential was 1.2 nm. The long-range electrostatic interaction was calculated using the PME method.[6] The LINCS algorithm constrained bond vibrations.[7] The OPLS-AA force field[8] describes the interaction in phospholipid molecules and polymers with SPC/E[9] model for water.

Fluorescence Activated Cell Sorting (FACS): Flow cytometry measurements were performed on a MoFlo Astrios EQs Sorter (Beckman Coulter, US) with 1.5×10^5 events per sample irradiating at 488 nm with a detection range of 513-526 nm. For quantification, median fluorescence intensity was analyzed with the FlowJo software (Tree Star, Inc., USA).

Characterization of i-combisome and membrane properties

Analysis of the lateral mobility: The diffusion coefficient was determined from the analysis of fluorescence recovery after photobleaching (FRAP) according to a previously reported protocol.[10] Vesicles were prepared by thin-film rehydration using 0.1 mol% of 16:0 Liss-Rhod PE as fluorescent probe. Free floating vesicles were recorded using a 561 nm laser in a 256 x 256 pixel frame in the bidirectional scan mode at 1800 Hz with the pinhole at 1 Airy unit. We recorded 10 pre-bleach frames at an attenuated laser power of 1%. Using a nominal bleaching radius of r_n = 2.5 μm bleaching was performed for 10 frames using maximum laser power. Afterwards, the fluorescence recovery was recorded at an attenuated laser power of 1% for several seconds.

First, we determined the effective bleaching radius r_e from the intensity line profile across the center of the first bleaching spot by using the fitting equation

$$f(x) = 1 - K\, exp \frac{-2x^2}{r_e^2}$$

Where K is the bleaching depth. From the normalized intensity FRAP curve we obtained the time $t_{1/2}$ until half of the fluorescence intensity $F_{1/2}$ recovered by using the equation

$$F_{1/2} = \frac{F_0 + F_\infty}{2}$$

F_0 is the fluorescence intensity in the bleached spot of the first frame after bleaching and F_∞ the intensity after recovery. With the obtained values for r_e and $t_{1/2}$ we determined the diffusion coefficients according to

$$D = \frac{r_e^2 + r_n^2}{8\, \tau_{1/2}}$$

Fluctuation analysis: We analyzed the undulations of the i-combisomes membranes by CLSM. The i-combisomes were prepared by thin-film rehydration with 0.1 mol% nile red. 16 µl of vesicles solution in water (1mg·mL^{-1}) were mixed with 4 µl 10 mM HEPES to deflate the vesicles and allow the fluctuation. This was followed by observed in CLSM. Images were taken with a resolution of 512x512 pixels at a scan speed of 1000 Hz. The analysis of the contours is discussed in the Supporting Results section (*vide infra*)

Membrane permeability to Co^{2+} ions and thermal stability of liposomes and i-combisomes: For observation in CLSM i-combisomes and DLPC liposomes labeled with 0.1 mol% nile red were prepared by thin-film rehydration in an aqueous solution of calcein ($c_{amphiphiles}$ = 1 mg·mL^{-1}, $c_{calcein}$ = 0.03 mg·mL^{-1}, 48 µM). For FACS vesicles were prepared by injecting a iCP or liposome solution ($c_{amphiphiles}$ = 0.5 mg·mL^{-1}) to an aqueous solution of calcein. To quench the calcein fluorescence outside the i-combisomes' lumen we added 2 µL of a $CoCl_2 \cdot 6H_2O$ in water (c = 0.5 mg·mL^{-1}, c_{Co2+} = 13 µM) to 300 µL vesicle dispersion. To assess the temperature stability, 50 µl of vesicle dispersion was heated to 80 °C for 1 h using a water bath. Vesicles were investigated by monitoring the fluorescence stemming from the membrane (nile red) and from the lumen (calcein) using CLSM while the object fluorescence intensity in FACS before and after addition of Co^{2+}, and after heating.

Co-assembly of iCPs with lipids: To assess the compatibility of the i-combisomes with lipids, vesicles were prepared by thin-film rehydration from i-CPs with different amounts of (Rhod-PE 0.1 - 40 mol%). As a control sample rhodamine labeled $DP_{85}N_{43}DS_{100}$ i-CP was co-assembled via thin-film rehydration with 40 mol% 16:0 NBD PE lipid.

Co-assembly of iCPs with glycolipids: $DP_{30}N_{30}DS_{100}$ i-CP was co-assembled with 20 mol% 16:0-18:1 DG glucose lipid and 0.1 mol% nile red via thin-film rehydration. The resulting vesicles were imaged by CLSM.

DNA loading: 20 µl of i-combisomes $D_{85}N_{43}DS_{100}$ were mixed with 5.27 µl (100µM, 5mol%) of single-stranded DNA labelled with Atto620 (3´-CTC GTA CGA GCC CCC C - Atto620 - 5´) and observed directly in CLSM.

Insertion of α-hemolysin: 5 µl of α-hemolysin in sodium citrate buffer (20 µM) were added to 15 µl of iCPs dispersion (c = 10 mg·mL^{-1}) with encapsulated calcein and Co^{2+} in the outer solution. The vesicle dispersion was visualized by CLSM after 1 h of the initial injection.

Fusion of i-combisomes with liposomes: For this studies all vesicles were prepared by thin film rehydration. Firstly, we assessed the fusion of cationic i-combisomes and anionic liposomes. The cationic i-combisomes were formed by $DP_{85}N_{43}DS_{72}$ i-CP with 0.1 mol% Rhod-PE. The anionic liposomes were assembled from DLPC and DLPG in a 4:1 molar ratio using 0.1 mol% NBD-PG. The same volume of liposomes was mixed with i-combisomes and immediately observed by CLSM. The observation continue for at least after 6 h incubation.
Then we assessed whether fusion could occur between i-combisomes and liposomes assembled from electroneutral building blocks. The liposomes were formed from DLPC with 0.1 mol% NBD-PC and the i-combisomes from $DP_{85}N_{43}DS_{100}$ with 0.1 mol% Rhod-PE. The vesicle fusion was studies as described in paragraph above.

Fusion of i-combisomes with bacteria membrane: We studied whether the i-combisomes could fuse with a living matter. For this we utilized as a model organism *E. coli* BL21 (DE3) gold cells. The bacteria were incubated in 5 mL LB-media (10 g·L^{-1} tryptone, 5 g·L^{-1} yeast extract, 10 g·L^{-1} NaCl, 0.1 mM kanamycin) overnight (14 h, 37 °C, 180 RPM, 70% humidity). 2 mL of the culture (diluted with LB media to OD 3.5) were centrifuged at 11000 x g for 30 s at 4 °C (Centrifuge 5424 R, Eppendorf, Germany) and resuspended in tap water (filtered, 0.2 µm).
iCPs (1 mg·mL^{-1}) labeled with 0.1 mol% nile red were mixed with bacteria 9:1 v/v and observed immediately in CLSM and for a course of 10 h.

Assembly of a model prototissue: A surrogate of the extracellular matrix was prepared by the surface-initiated fibrin network formation in the presence of i-combisomes. This method developed by Riedel allows for the initiation and nucleation of fibrin fiber formation from the surface a substrate. [11] Firstly, fibrinogen was adsorbed on microscope cover glasses (24 x 60 mm) from a solution human fibrinogen (10 mg·mL^{-1} in Tris-buffer pH = 7.4) for 1 h. Afterwards, the surface was rinsed with Tris-buffer and adsorbed fibrinogen was activated by putting 200 µL of the thrombin solution for 2 h. The surface was rinsed with Tris-buffer so that only the thrombin bound to the adsorbed fibrinogen remained. In the last step, 200 µL of a solution of fibrinogen (1 mg·mL^{-1}), FITC-labeled fibrinogen (1 wt%) and i-combisomes ($DP_{30}N_{30}DS_{100}$, 1 mg·mL^{-1}, 178 µL) in MilliQ water were added onto the surface overnight. This allowed the fibrin network formation by a catalytic reaction between surface-bound thrombin and fibrinogen in solution. The formation of the fibrin network in the presence of i-combisomes led to the incorporation of the i-combisomes in the network.

2. Supplementary Results

2.1 Synthesis of iCPs

CBAA and DMAPAA were polymerized by SET-LRP in water at 0 °C using a water-soluble tertiary bromide initiator (Figure S1 d). As the catalyst, Cu(0)-particles were directly generated from Cu(II)Br_2/Me_6TREN in-situ by reduction with $NaBH_4$. We optimized the polymerization conditions to yield quantitative conversions and narrowly dispersed polymers (Figure S2, b). Kinetic studies of the optimized polymerization revealed a first-order kinetic behavior (Figure S2, a) demonstrating uniform growth. The kinetic profile further indicates that the probability of incorporating either CBAA or DMAPAA into the growing chain is similar and remains constant throughout the polymerization. This results in a copolymer where both monomers are statistically distributed along the chain.

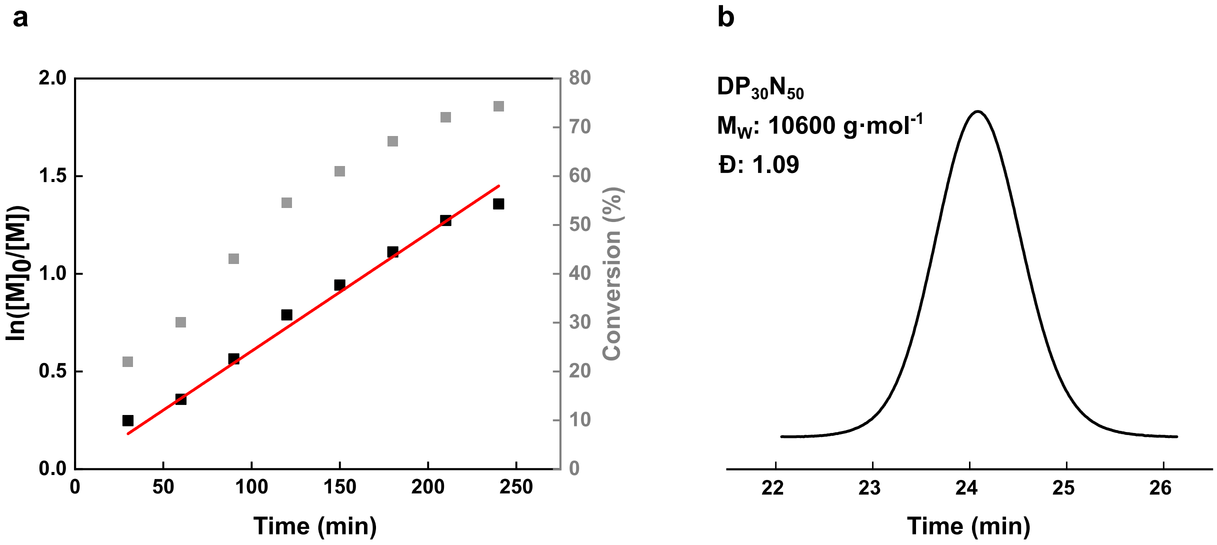

Figure S2. (a) Conversion and ln($[M]_0$/[M]) vs time kinetic plots for $DP_{30}N_{50}$. (b) SEC elugram for $DP_{30}N_{50}$.

Molecular characterization of all synthesized iCP-backbones (Table S2) were performed by SEC, ^{1}H-NMR and ^{13}C-NMR. A representative NMR is shown for $DP_{30}N_{30}DS_{100}$ in Figure S3 and S4. The composition, as well as the DP of the purified copolymer, were calculated from ^{1}H-NMR using the initiator signal **17+18** (1.03 ppm, 6H, 2x -CH3) together with signal **9** of CBAA (3.43 ppm, 2H, CH2) and signal **14+15** of DMAPAA (2.75 ppm, 6H, 2x –CH3). The resulting polymer composition of 70% CBAA and 30% DMAPAA with DP = 30 is in excellent agreement with the monomer feed ratio and the target DP. SEC-analysis in water revealed a narrowly dispersed and monomodal molecular weight distribution (Đ < 1.1, Table S2).

Table S2. Molecular characterization of the synthesized iCP's backbones.

Entry	Name	CBAA:DMAPAA	$DP^{NMR\ a}$	$M_n^{NMR\ a}$ (g·mol^{-1})	$M_w^{SEC\ b}$ (g·mol^{-1})	$Đ^{SEC\ b}$
1	$DP_{30}N_{15}$	85:15	35	6520	9400	1.09
2	$DP_{30}N_{30}$	70:30	30	6200	11400	1.09
3	$DP_{30}N_{50}$	50:50	30	5770	10600	1.08
4	$DP_{30}N_{70}$	30:70	30	5340	10700	1.08
5	$DP_{85}N_{43}$	57:43	89	17560	53100	1.65
6	$DP_{85}N_{57}$	43:57	98	18350	72700	1.81
7	$DP_{85}N_{70}$	29:71	72	12750	55700	1.60
8	$DP_{400}N_{48}$	52:48	405	78450	223000	2.01

a) DP and Mn calculated from 1H-NMR of the purified polymer, b) SEC in water with linear poly(PEO) standards and RI-detector

A library of iCPs with precise structural variations was prepared by the addition of DDP in chloroform to a solution of the copolymer backbone in methanol, resulting in the ionically comb-like structure (Figure S1f). Molecular characterization of all synthesized iCPs (Table S3) were performed by ^{1}H-NMR and ^{13}C-NMR in a mixture of $CDCl_3$ and MeOD (1:1). A typical NMR of $DP_{30}N_{30}DS_{100}$ is shown in Figure S5 and S6). We estimated the molecular weight of the iCPs based on the molecular weight of the backbone obtained by ^{1}H-NMR and the amount of complexed DDP. As a result, we obtained macromolecular amphiphiles from 8.5 $10^3$to 1.61 10^5 g·mol^{-1} that self-assembled in water in supramolecular structures.

Table S3. Library of iCPs

Entry	Name	DP^{NMR}	X_{DMAPAA} [%]	DS	DDP [%]	M_n^{NMR}x10^{-3} [g·mol^{-1}]
1	$DP_{30}N_{15}DS_{100}$	30	15	100	15	8.5
2	$DP_{30}N_{30}DS_{100}$	30	30	100	30	10.1
3	$DP_{30}N_{50}DS_{80}$	30	50	80	40	9.7
4	$DP_{30}N_{50}DS_{100}$	30	50	100	50	12.3
5	$DP_{30}N_{70}DS_{72}$	30	70	72	50	11.9
6	$DP_{30}N_{70}DS_{100}$	30	70	100	70	14.5
7	$DP_{85}N_{43}DS_{50}$	89	43	50	21	26.1
8	$DP_{85}N_{43}DS_{70}$	89	43	70	30	29.2
8	$DP_{85}N_{43}DS_{100}$	89	43	100	43	34.2
9	$DP_{85}N_{57}DS_{80}$	98	57	80	45	37.5
10	$DP_{85}N_{57}DS_{100}$	98	57	100	57	42.6
11	$DP_{85}N_{70}DS_{72}$	71	70	72	50	28.6
12	$DP_{85}N_{70}DS_{100}$	71	70	100	70	34.2
13	$DP_{400}N_{48}DS_{100}$	400	48	100	48	160.9

All values were calculated from 1H-NMR.

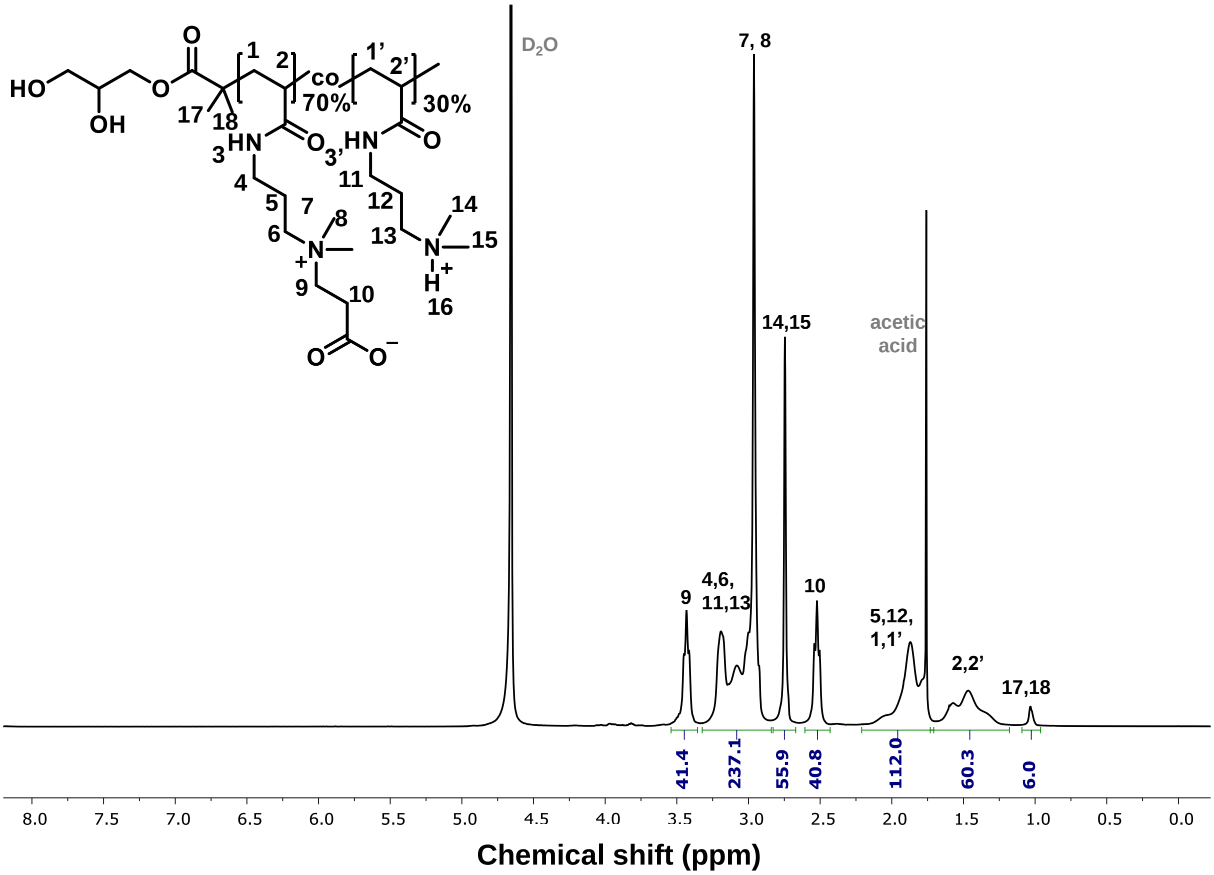

Figure S3. ^{1}H-NMR of the synthesized poly(CBAA-co-DMAPAA) ($DP_{30}N_{50}$) in D_2O.

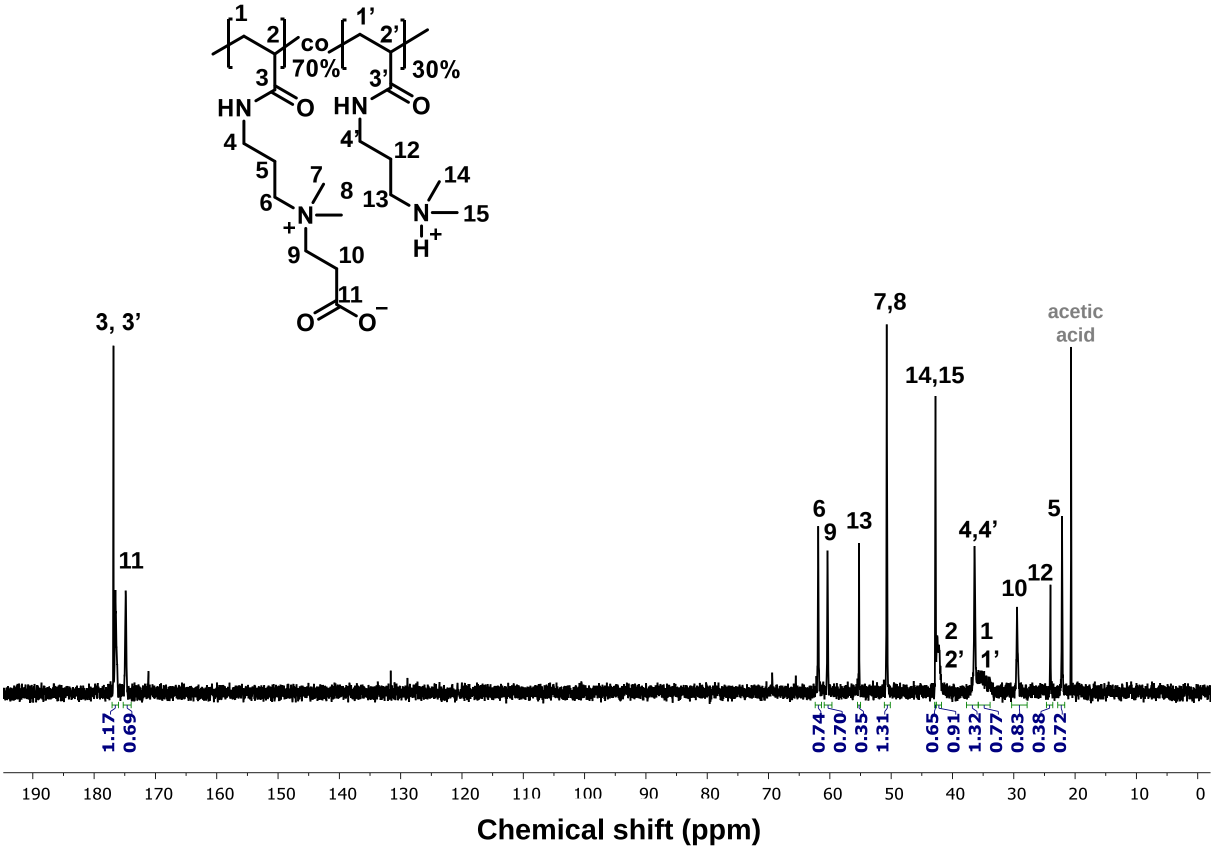

Figure S4. ^{13}C-NMR of the synthesized poly(CBAA-co-DMAPAA) ($DP_{30}N_{50}$) in D_2O.

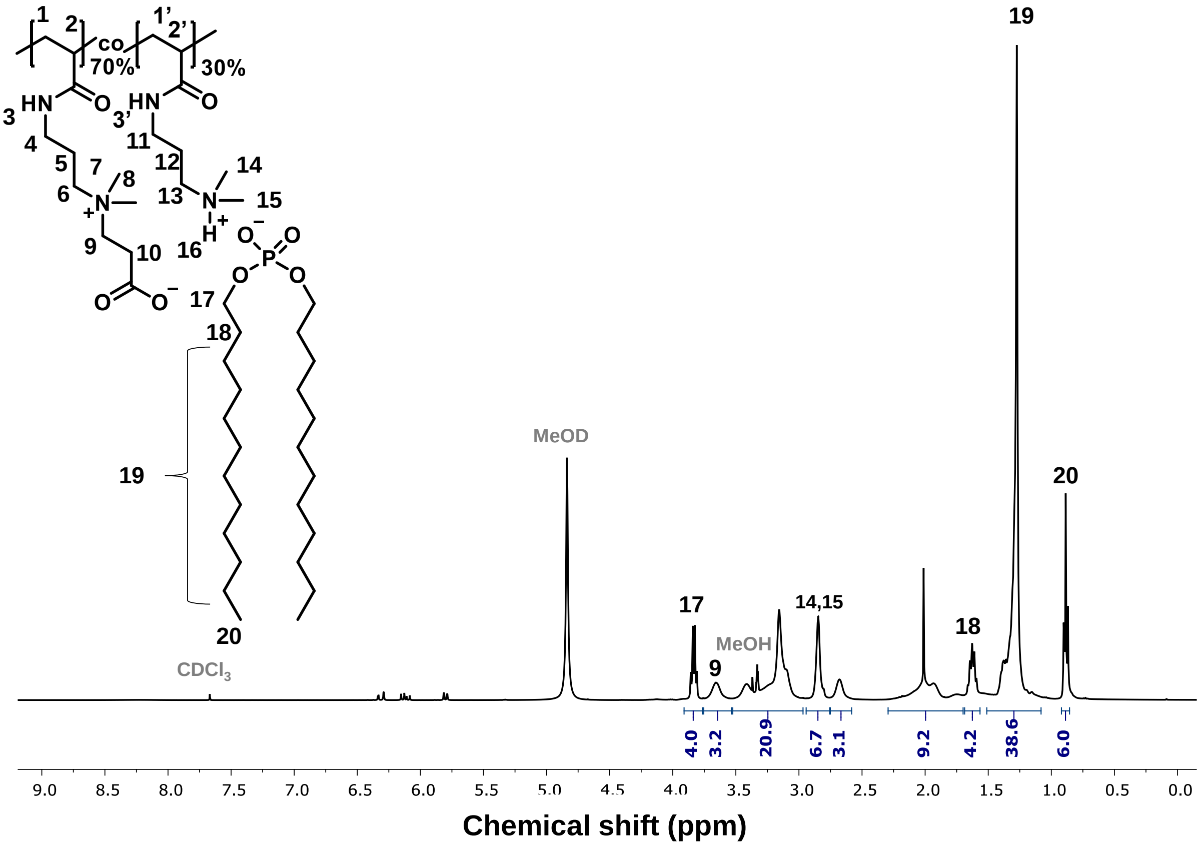

Figure S5. ^{1}H-NMR of the iCP $DP_{30}N_{30}DS_{100}$ formed by complexation of $DP_{30}N_{30}$ poly(CBAA-*co*-DMAPAA) copolymer with DDP in $CDCl_3$:MeOD (1:1).

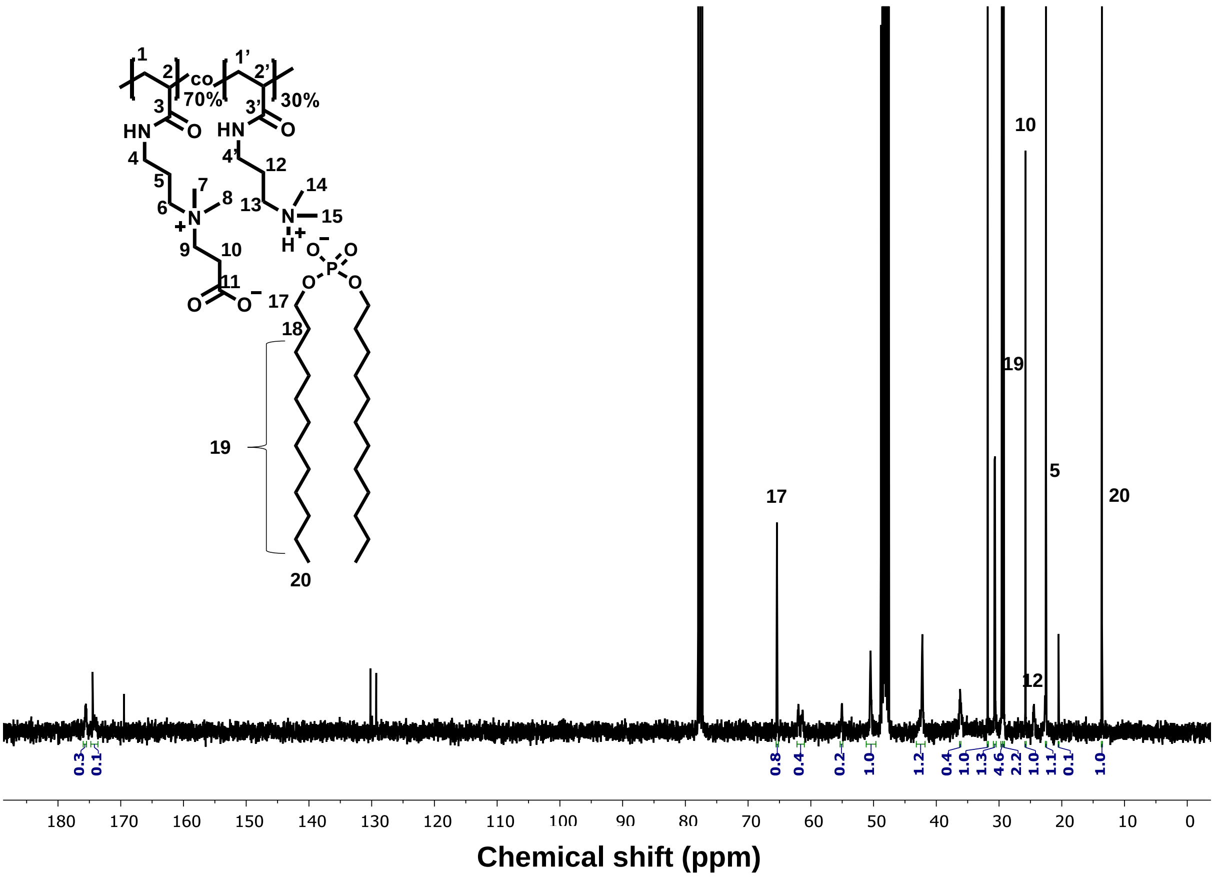

Figure S6. ^{13}C-NMR of the iCP $DP_{30}N_{30}DS_{100}$ in $CDCl_3$:MeOD (1:1).

2.2 Self-assembly of i-CPs

We studied how the molecular structure of the iCPs controls their self-assembly in water into supramolecular structures (Figure S8). We proposed that the structure formation can be predicted by the packing parameter of an equivalent amphiphilic repeating unit (ARU) of the polymer similar to a low molecular weight amphiphile (Figure S7). Structural variation in the iCPs was created from DP = 30 and DP = 85, where we systematically varied *N* and *DS* to tailor the density of hydrophobic alky tails in the resulting iCPs. Figure S8 summarizes the different structures emerging for DP = 30. In the CLSM, giant unilamellar i-combisomes were observed in the range of ρ_{DDP} 30 – 70%. For ρ_{DDP} = 15 ($DP_{30}N_{15}DS_{100}$), small aggregates with no distinct shape or structure were observed. The dynamic light scattering (DLS) shown in Figure S8 and cryo-TEM revealed the presence of micelles as the predominant structure.

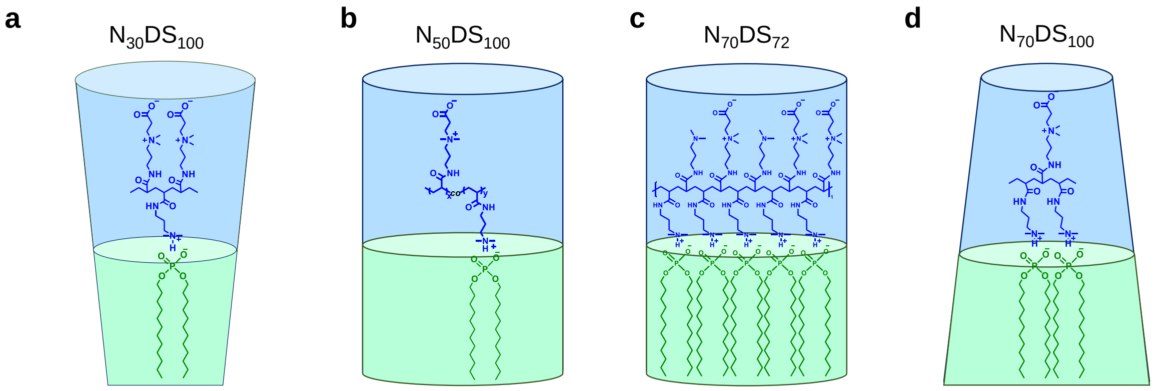

Figure S7. Variation in the packing shape of the amphiphilic repeating unit of the iCPs depending on *N*, *DS*, and ρ_{DDP}. The amphiphilic repeating unit is independent of the *DP*.

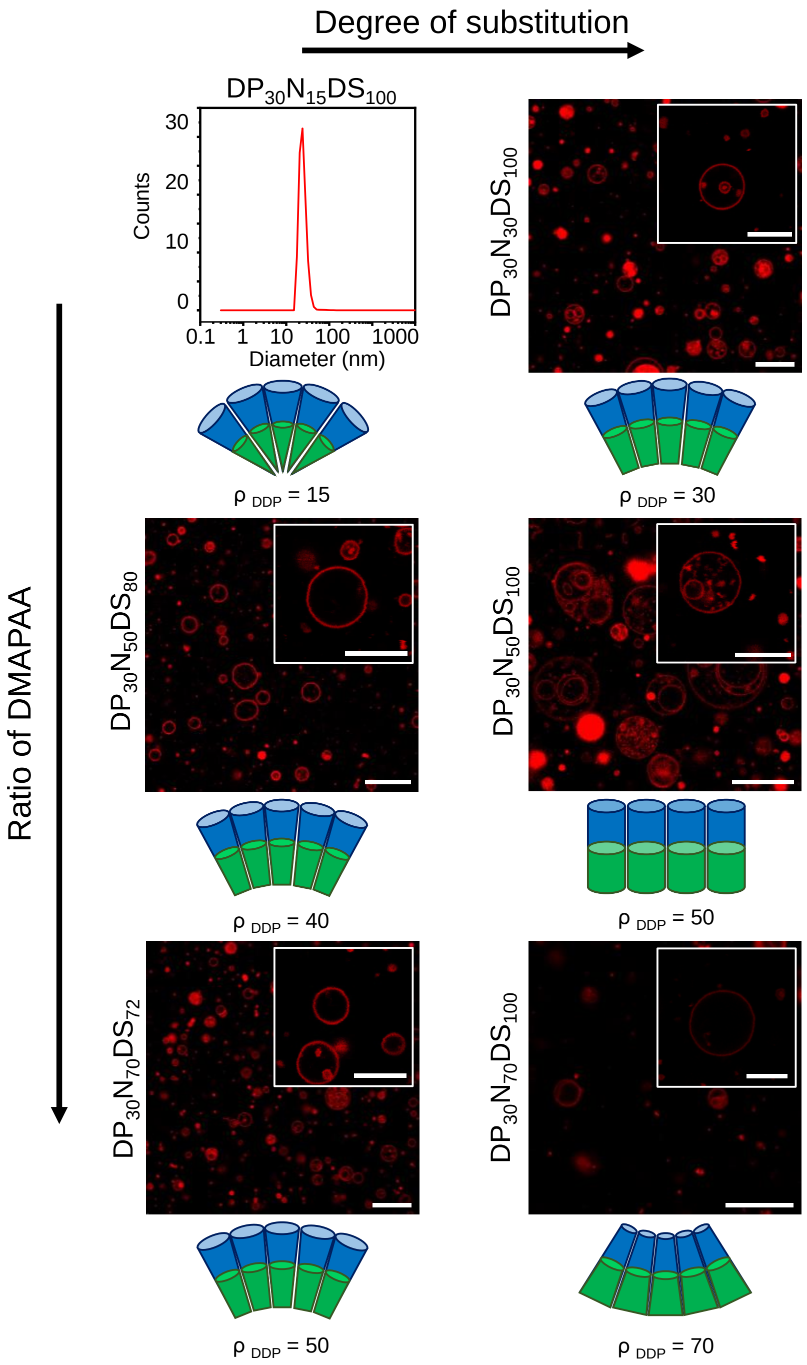

Figure S8. Self-assembly of a library of iCPs with *DP* = 30 in water studied by CLSM. The assembly was studied as a function of ρ_{DDP} which controls the packing parameter of an equivalent repeating unit depicted below the respective confocal images. ρ_{DDP} was adjusted by varying *N* and *DS*. The insets show an image of a representative i-combisome. Scale bars are 20 µm for overview images and 10 µm for insets. No large assemblies were observed in $DP_{30}N_{15}DS_{100}$ using CLSM; thus, we studied it by dynamic light scattering ((a), number distribution) and cryo-TEM (Figure S9).

To explore the effect of the molecular weight of the macromolecular amphiphile on the structure formation, the iCP-backbone length was increased to DP = 400. Figure S9 shows the successful self-assembly of $DP_{400}N_{50}DS_{100}$ into GUVs.

Our results demonstrate that in i-combisomes, the self-assembly into bilayer vesicles is mainly controlled by ρ_{DDP} and largely independent of the molecular weight of the amphiphile, which is in stark contrast to traditional polymersomes from block copolymers.

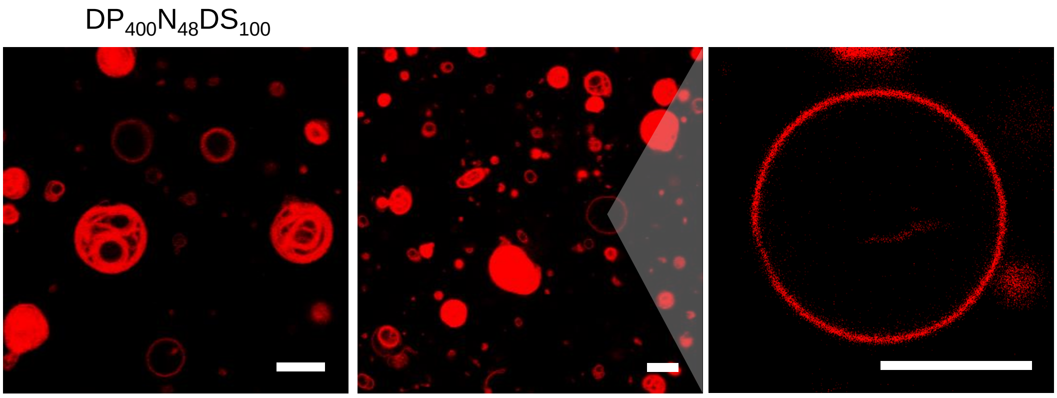

Figure S9. CLSM-image of GUVs from i-combisomes $DP_{400}N_{48}DS_{100}$. The scale bar is 10 µm.

Determination of the membrane thickness of i-combisomes (AFM and Cryo-TEM)

While in polymersomes from block copolymers the membrane thickness scales with the length of the hydrophobic block, cryo-TEM studies of the i-combisomes (DP_{30}, DP_{85}, DP_{400}) revealed that the membrane thickness was invariant regardless of the molecular weight of the amphiphile with biomimetic thickness *ca* 5 nm (Figure S10). The bilayer thickness was determined in ImageJ.

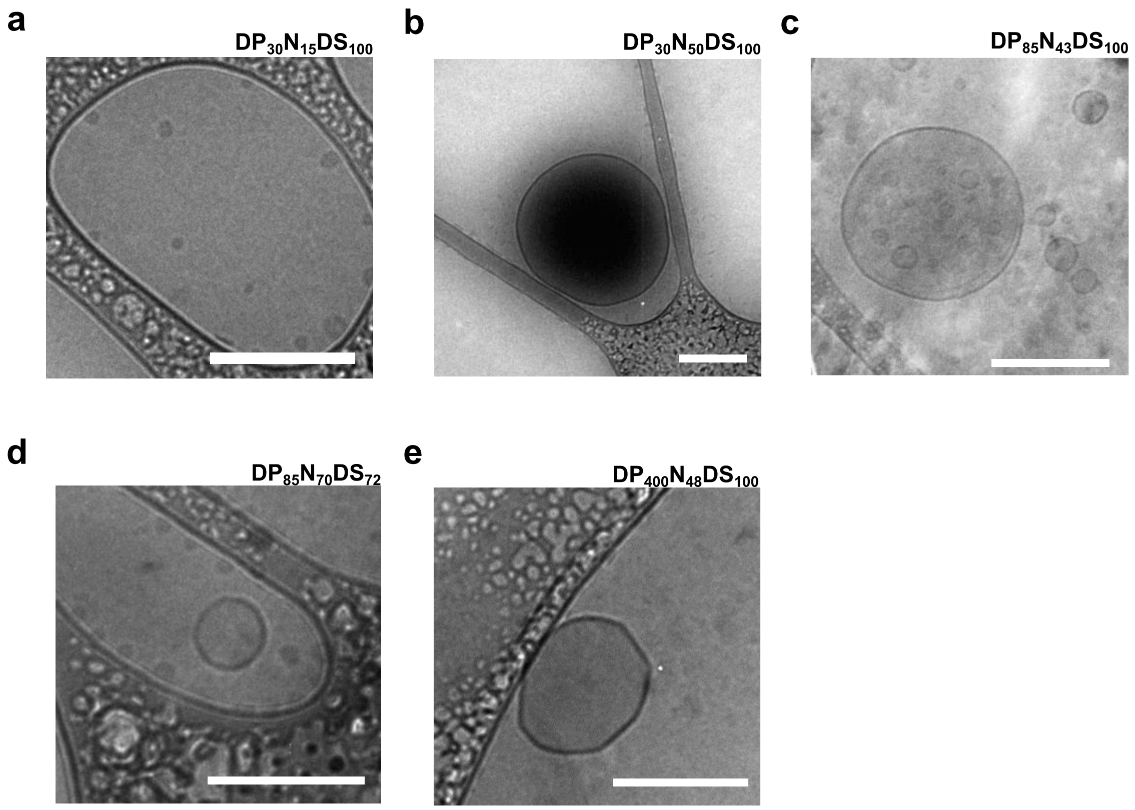

Figure S10. CryoTEM of resulting supramolecular assemblies of various iCPs in water (a) $DP_{30}N_{15}DS_{100}$ showing micelles formation as majoritarian aggregate type. (b-e) images of i-combisomes. Scale bars are 200 nm.

We utilized AFM microscopy also to investigate the thickness of bilayers deposited on mica. Figure S11 depicts bilayers of i-combisomes with varying *N* to determine whether this parameter could influence the thickness. AFM was recorded at 55% relative humidity for i-combisomes of $DP_{85}N_{43}DS_{100}$, $DP_{85}N_{57}DS_{100}$, $DP_{85}N_{70}DS_{100}$. Figure S11 shows the height images, phase images, and the respective height profiles along the lines indicated by the arrows in the height images. We could conclude from the height profiles that the bilayer thickness is invariant with *N* in the studied range. The uniform thickness obtained from the experimental cryo-TEM and AFM studies, combined with the atomistic molecular dynamic simulation (see

section simulation), indicates that the length of the hydrophobic tails almost exclusively controls the membrane thickness.

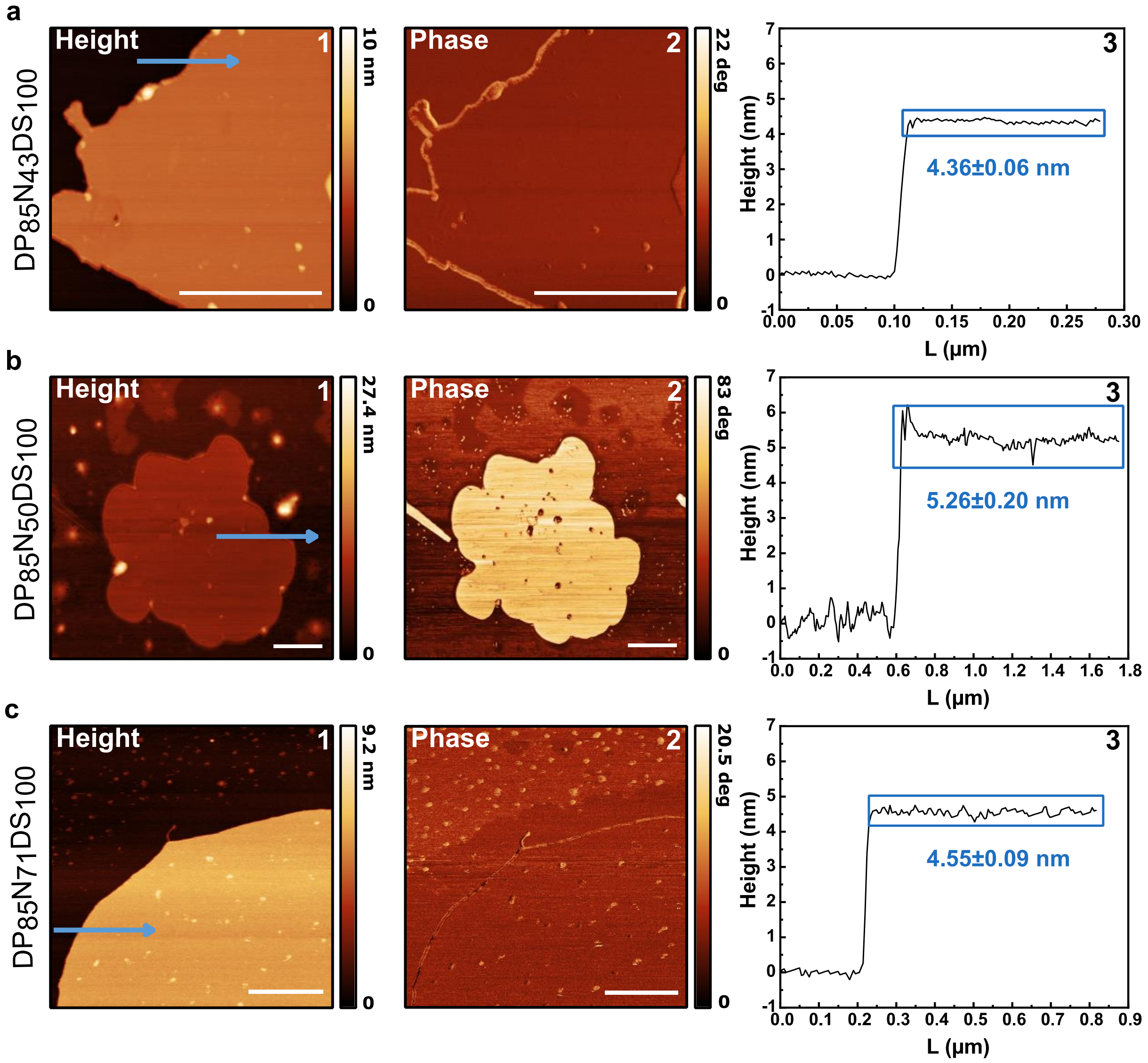

Figure S11. Study of the correlation between *N* (a-c) and i-combisome bilayer thickness by AFM displaying the topography (1), phase (2), and the height profile (3) taken along the blue arrow in (1).

Faceted i-combisomes

Cryo-TEM revealed the presence of some small fraction of vesicles with markedly faceted shape. This observation was prominent in smaller i-combisomes <100 nm for DP = 30 and DP = 85, while for DP = 400 no faceted structures were observed (Figure S12). The faceting was most pronounced in DP30, with a nearly square structure and sharp edges.

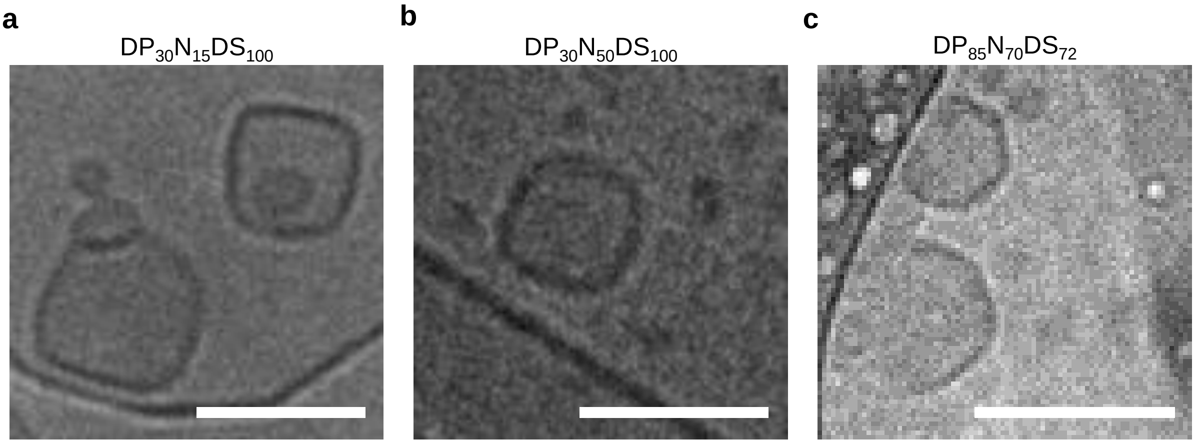

Figure S12: cryo-TEM images of facetted vesicles obtained from (a) $DP_{30}N_{15}DS_{100}$, (b) $DP_{30}N_{50}DS_{100}$, (c) $DP_{85}N_{70}DS_{72}$, Scale bars: 100 nm

Atomistic molecular dynamic simulations

Atomistic dynamic simulations were performed to provide insight into the i-combisome structure, applying the protocol described in the method section (*Atomistic molecular dynamics simulation*). To obtain a phospholipid bilayer, we arranged 1900 surfactant molecules in hexagonal close-packed order in the midplane of a 23208 nm^3 sized box. The number of surfactant molecules remained constant in all simulated systems. First, sodium atoms were added to the bilayer to neutralize the charge of the system, followed by equilibration of the bilayer, which was well equilibrated after 100 ns. Then the z-direction of the box was extended to 20 nm, and polymer backbones were added. The sodium atoms were also required as a counterion because in most cases the remainder of the division of the number of phospholipids by the number of polymer charges was not an integer. Table S4 summarizes the simulated samples.

Table S4: The respective simulated systems are shown, including their essential constituents. The number of polymers corresponds to the charge density and the degree of substitution.

i-Combisome	Number of DDP	Number of iCPs
$DP_{30}N_{50}DS_{80}$	1900	100
$DP_{30}N_{50}DS_{100}$	1900	126
$DP_{30}N_{70}DS_{100}$	1900	100

The iCP-backbones were considered monodisperse in the simulation. Therefore, the iCP-backbones were placed in equal quantities on both sides of the bilayer. The simulation for the bilayer with adsorbed polymer ran for at least 100 ns. The last 10 ns of the simulated trajectories were used to analyze the data. Figure S13 shows the final state of the simulated i-combisome membrane. The membrane consists of three zones containing a hydrophobic core domain of DDP onto which the hydrophilic iCP backbone adsorbs. The snapshots reveal a stretched conformation of the iCPs that appear to be ordered in the membrane in a nematic manner.

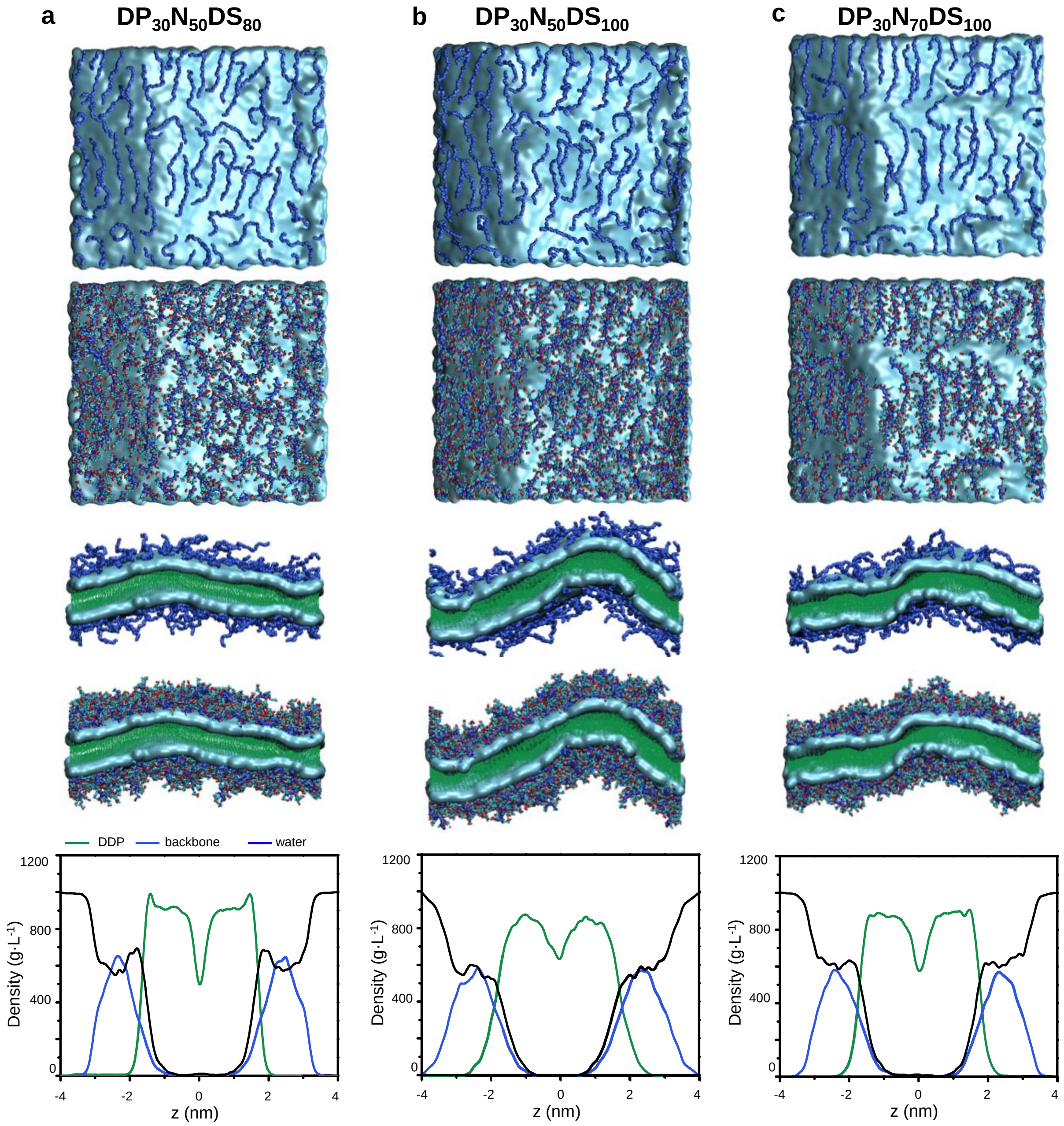

Figure S13: Atomistic molecular dynamic simulations of varying i-combisome composition. Snapshot of simulated bilayers of $DP_{30}N_{50}DS_{80}$ (a), $DP_{30}N_{50}DS_{100}$ (b), and $DP_{30}N_{70}DS_{100}$ (c). Top view images show the backbones (no explicit side groups and with the explicit) organized at the interface with water. The side views (with and without sidegroups) display the organization of the membrane with the backbone restricted to the interface with water. Density profile of DDP (green), polymer backbone (blue), and water (black) in the respective i-combisome.

For the calculation of the density profiles the xy-plane was divided into small squares with the z-plane set as the normal line. The defined squares contained a flat section of the bilayer. From each square, the respective density profile was calculated and then averaged over all squares. The density profiles show very high similarities despite the disparity of the molecular compositions. For all the bilayers the hydrophobic domain was approximately 4 nm and almost no penetration of water or polymer backbone occurred. Polymer zones on each side of the hydrophobic domain were highly hydrated, as evidenced by water penetration. Remarkably, 90% of the polymer mass was found in a thin layer of just 1.5 nm. Such a thin layer can only be achieved when the backbone is stretched and confined to a 2D conformation at the membrane water interface.

The thickness of the bilayers was calculated as the average distances between polymer slices. The position of the slices was defines as the volume that contains 90% of polymer mass (Figure 3 h, in the Manuscript). The obtained bilayer thickness is between 5-6 nm, in agreement with the experimental results.

The snapshots showed that the polymer acquired a rod-like conformation with a preferential organization in the bilayer displaying a nematic type of order. A similar observation was for the DDP, which also seems to adopt a L_α liquid crystalline. The organization of the backbone and DDP can be estimated by an average orientational order parameter S for each part (Figure 3 i).

The order in the bilayer was further quantified by the deuterium order parameter S_{CD} according to the following equation:

$$S_{CD} = \langle \frac{3\cos\theta^2 - 1}{2} \rangle$$

where Θ is the angle between bilayer normal and the vector lined from carbon atom to hydrogen atom in an alkyl tail of phospholipid. Figure S14 shows the carbon atoms that were used for the calculation. The second tail was numbered similar to the first tail.

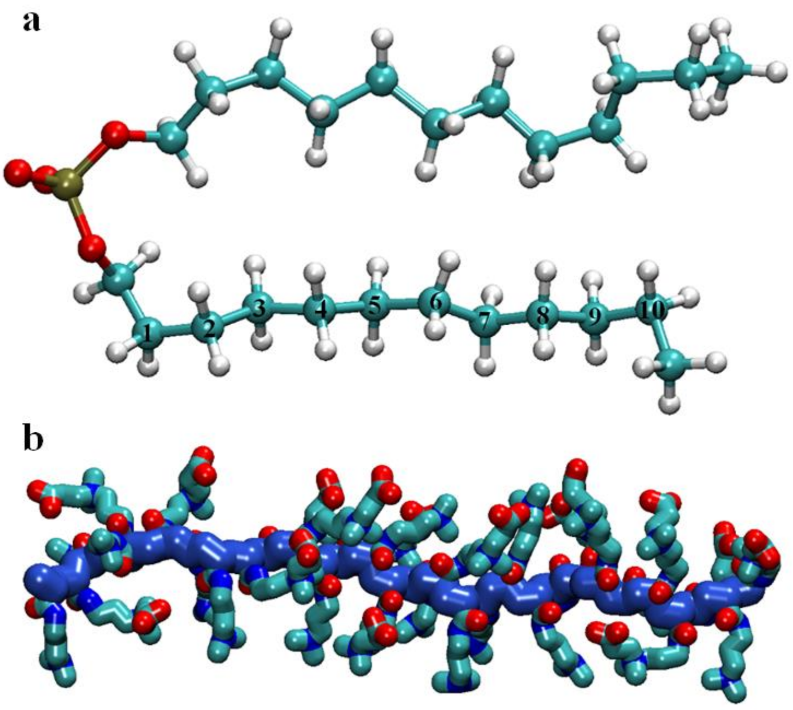

Figure S14. (a) Atomistic representation of DDP used for the simulations. The black numbers denote the atom numbers used for the calculation of S_{CD} (Figure 3 j, Manuscript). (b) Typical conformation was observed in the simulation after equilibration. The backbone acquired a rod conformation. The backbone corresponds to $DP_{30}N_{50}DS_{80}$ system (hydrogen atoms are not shown).

We also calculated the ratio of R_g/R_g^{rod} where R_g and R_g^{rod} are the radii of gyration for the backbone at the bilayer and for an equivalent rod. The radius of gyration were calculated according to the following equation:

$$R_g = \sqrt{\frac{\sum_{i=1}^{N} m_i (r_i - r_c)^2}{\sum_{i=1}^{N} m_i}}$$

where m_i and r_i are the mass and the coordinate of the i-th atom, and r_c defines a coordinate of the center of mass.

We found that R_g/R_g^{rod} was between 0.95 and 0.98 for all compositions. This demonstrates that the backbones were stiff rods. Figure 14 b display an atomistic 3D representation in close agreement with the R_g/R_g^{rod}.

2.3 i-Combisomes: a biomimetic liposome-polymersome chimera

FRAP of i-combisomes in a supported lipid bilayer

We also determined the *D* for supported bilayers of i-combisomes. We studied the diffusion of fluorescently-labeled lipid (Rhod-PE) in the i-combisome membrane and of the backbone itself by labeling the iCP with rhodamine. For the former, we formed vesicles of $DP_{85}N_{43}DS_{100}$ with 0.1 mol% of Rhod-PE while the latter was assembled from Rhod-iCP, $DP_{85}N_{43}DS_{100}$. The supported bilayers were prepared by depositing 20 μL of a vesicle dispersion, prepared by thin-film rehydration, into a CLSM observation chamber and incubated for 1 h at room temperature. Using the methodology described in methods, D was measured by FRAP analysis on the fluorescent supported bilayer. Remarkably, only a slight decrease in *D* was observed for Rhod-iCP 2.4 $\mu m \cdot s^{-1}$ compared to Rhod-DOPE 3.0 $\mu m \cdot s^{-1}$ despite the difference in size. *D* was lower than for the vesicles. However, it remains very high compared to polymersomes and in the range of lipids, indicating similar membrane dynamics.[12]

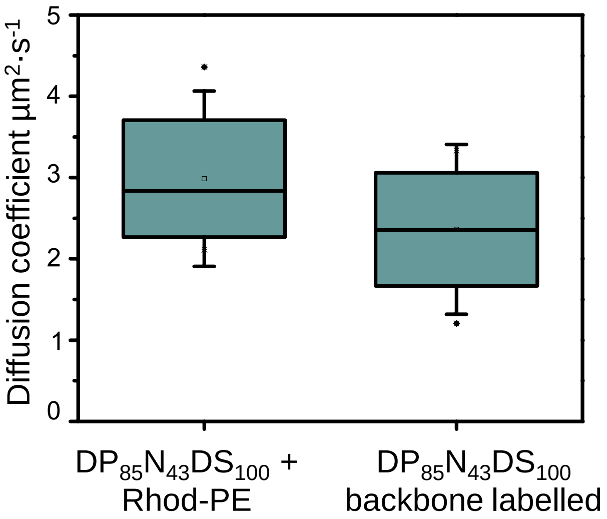

Figure S15. Boxplots for the diffusion coefficients of Rhod-PE in supported bilayers of $DP_{85}N_{43}DS_{100}$ and for the Rhod-labelled backbone of $DP_{85}N_{43}DS_{100}$. Boxes were generated from ten data points and contain the 25th to the 75th percentile of each data set. The line represents the median while an open rectangle indicates the average. The whiskers show the standard deviation while the outliers are displayed outside of the whiskers.

Analysis of membrane fluctuations

The flexibility of vesicles is partly reflected in the ability of their membranes to fluctuate. The molecular arrangements within the i-combisome membrane enable soft membranes similar to those in liposomes. CLSM-imaging of deflated i-combisomes showed that the membranes were constantly undulating. To assess the fluctuation, single CLSM images were processed using a custom-made script in python following a general procedure.[13] Objects not corresponding to the studied vesicle were removed before further processing using Fiji ImageJ.[14] A background image was created for each sample by convolution of a Gaussian kernel (window size of 61 pixels, width $\sigma = 10$ pixels). This background was subtracted from the original image. The obtained negative pixels were set to zero. A second Gaussian kernel (window size of 21 pixels, width $\sigma = 3$ pixels) was applied to smooth the image. A binary image was created from the results by applying a threshold calculated following Otsu´s method.[15] Confluent images with a predefined size range were selected from the binary images. The size range was typically ranging from 500-15000 pixels area for each image. The center of the mass defined the center of the object. The pixel coordinates were transformed to polar coordinates and angles relative to the center of the object. An angle range was defined from 0 to 2π rad with 100 data points. In each of the defined angle bins, the maximum was identified, then a local straight line was fitted in both directions from the maximum and the crossing point used as the corresponding radius.[16] The exported profiles were further processed using R. The mean radius was subtracted from each data set ($\Delta r = r - \langle r \rangle$)., which allowed to compare the angular profiles. By shifting of $\Delta r(\varphi)$ the zero angle was set to the maximum of the curve (main axis of a prolate) for each vesicle. Afterwards $\Delta r(\varphi)$ was fitted with a series of cosine functions with integer harmonics where φ is the angle obtained from the data set:

$$\Delta r = \sum_{i=1}^{N} \alpha_i \cdot \cos(i\varphi)$$

In the next step the shape contributions were removed to show the individual fluctuation independent of size and shape.[17] To remove the influence of shape the two first harmonics of the cosine series were subtracted generating $\Delta r(\varphi)$’ from $\Delta r(\varphi)$.

The analysis were performed for vesicles from a series of iCPs with precise structural variation, as well as for a lipid (DLPC) and for a block-copolymer (poly(BD_{87}-b-EO_{72}).

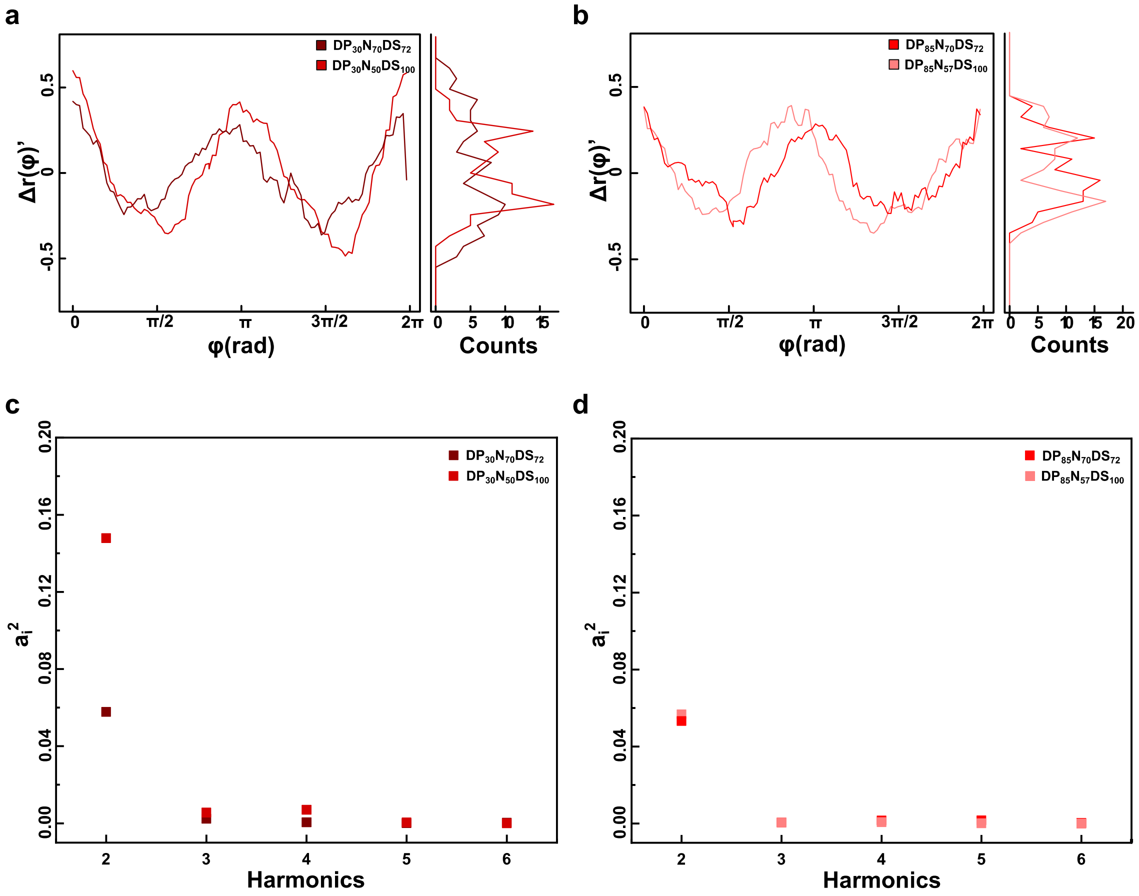

Figure S16. (a, b) Angular fluctuation of radii (Δr(φ)') after subtracting the first two harmonics of the cosine decomposition (left) and distribution of the fluctuations (right). (c, d) Amplitude of the harmonics.

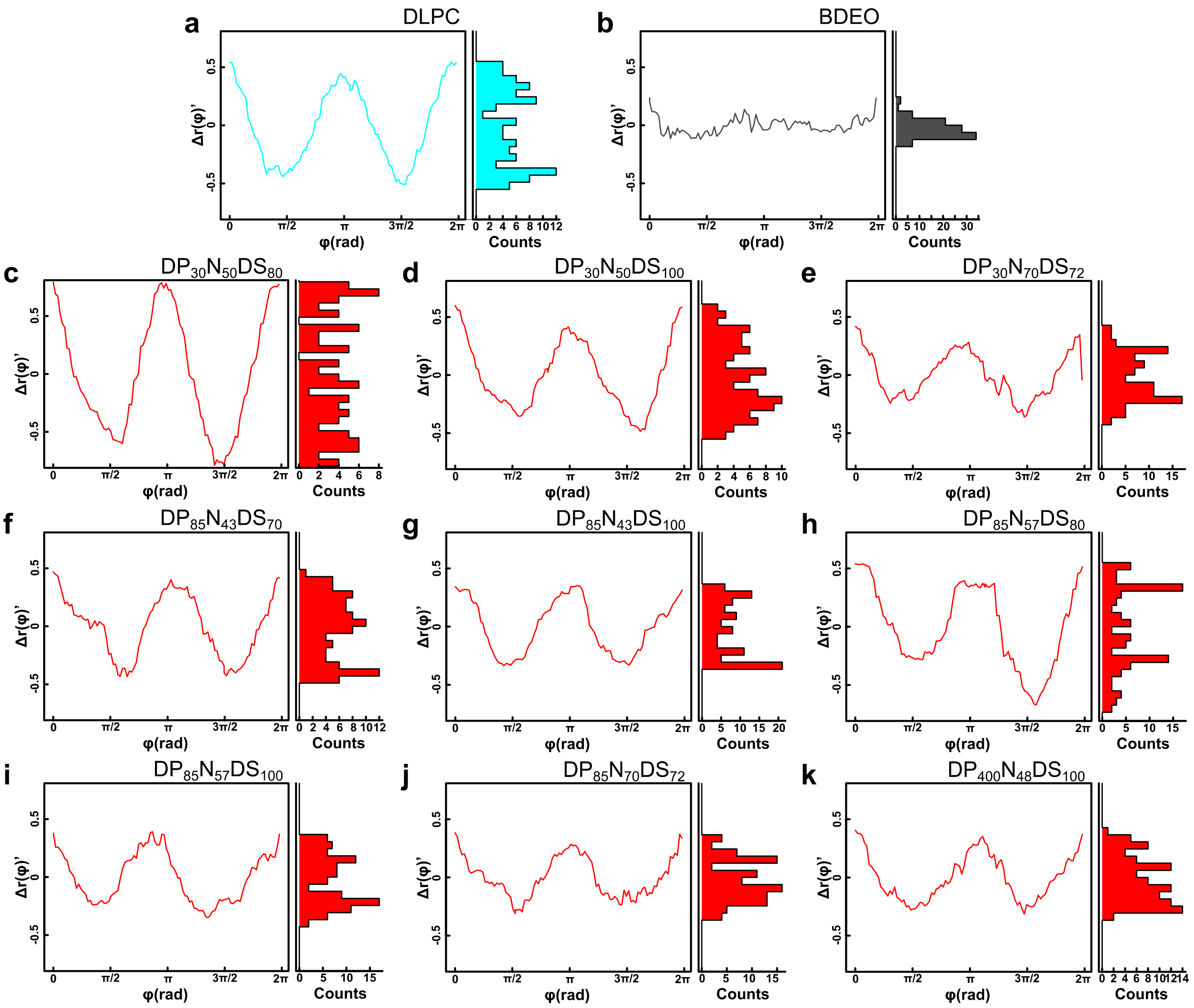

Figure S17. Angular fluctuation of radii (Δr(φ)') after subtracting the first two harmonics of the cosine decomposition (left) and distribution of the fluctuations (right) for (a) liposomes from DLPC, (b) polymersomes from BDEO (poly(BD_{87}-*b*-EO_{72}). and (c-k) for selected i-combisomes.

Thermal stability of i-combisomes

Figure S18 shows histograms of vesicle counts versus their associated fluorescence intensity for DLPC liposomes and $DP_{85}N_{43}DS_{100}$ i-combisomes. Vesicle dispersions before heating where no Co^{2+} was added (blue curve) and dispersions where it was added (red curve) show narrow monomodal distributions for DLPC and i-combisomes. In the case of liposomes addition of Co^{2+} led to a shift to lower intensities, possibly due to permeation of Co^{2+} into the vesicle lumen and the concomitant fluorescence quenching of calcein.[18] We did not observe such a shift for i-combisomes.

After thermal treatment (80 °C, 1 h) we observed significant broadening to lower and higher intensities for the histogram of DLPC liposomes (orange curve). A decrease in intensity is

obtained due to fluorescence quenching. We ascribe an increase in fluorescence intensity to vesicle aggregates that form upon heating, similar to our observations by CLSM (Figure 4 e in the Manuscript). On the other hand, the i-combisomes displayed a higher resilience towards the thermal treatment. The majority of the i-combisome population (80%) remained unchanged with about 20% displaying higher intensity which may be caused by fusion. In CLSM of the same i-combisomes after the treatment, we do not see broken or aggregated i-combisomes.

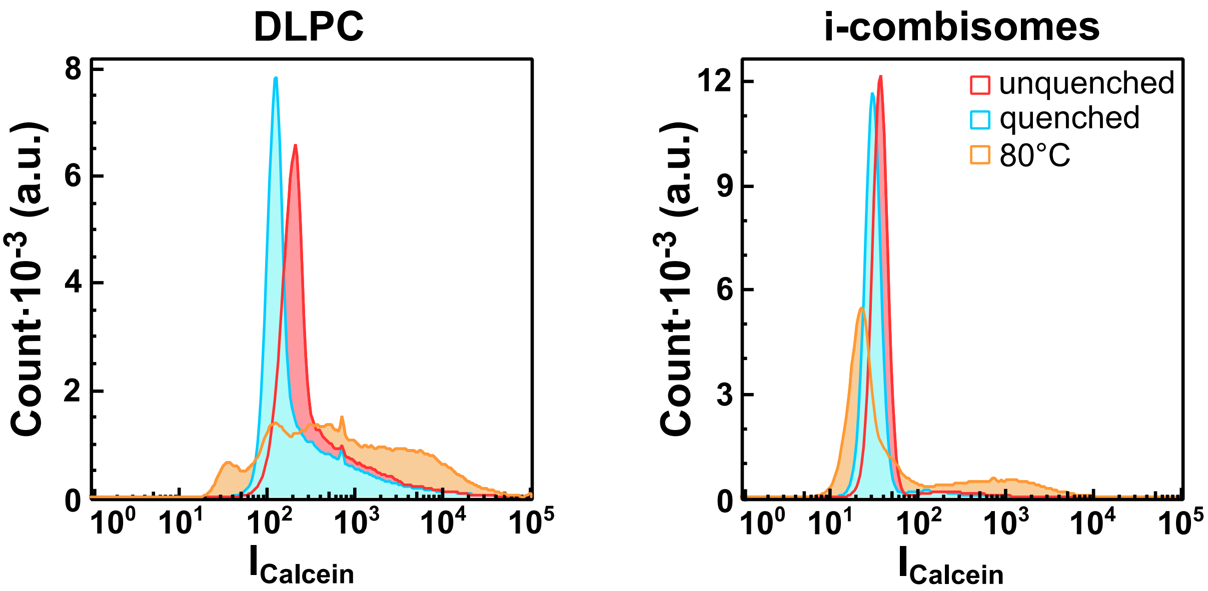

Figure S18. Histograms of vesicle counts vs. their associated fluorescence intensity for DLPC liposomes and i-combisomes ($DP_{85}N_{43}DS_{100}$) before (red) and after (blue) addition of Co^{2+} and after heating at 80°C for 1 h (orange).

2.4. Introducing biological functionality by co-assembly with biomolecules

Lipid – i-Combisome hybrids

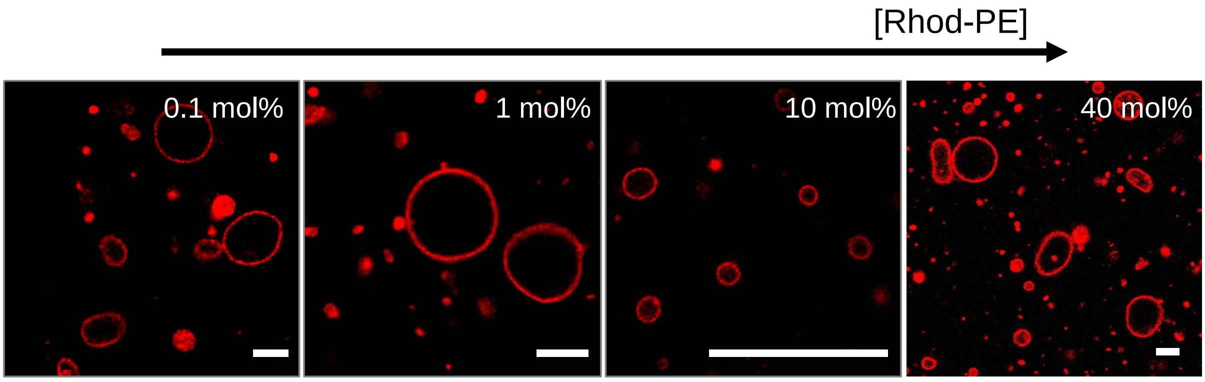

Figure S19. CLSM images of $DP_{85}N_{43}DS_{100}$ co-assembled with Rhod-PE in molar ratios ranging from 0.1 to 10 mol%. Scale bars: 5 µm.

Figure S19 shows CLSM images of i-combisome hybrids co-assembled from $DP_{85}N_{43}DS_{100}$ with different molar ratios of fluorescent rhodamine-labeled lipid. We observed successful co-assembly with no apparent phase separation using molar ratios of 0.1 mol% up to 40 mol% fluorescent lipid.

Raft-like microdomains

We performed Laurdan generalized polarization analysis using $DP_{85}N_{43}DS_{100}$ i-combisomes labelled with 1 mol% Laurdan by thin-film rehydration. We prepared three i-combisome hybrids where 20 mol% of DLPC (12:0 PC), DPPC (16:0 PC) and DSPC (18:0 PC) were additionally incorporated. As a control sample, we prepared i-combisomes with no additional lipids. We observed vesicles by CLSM using an attenuated (5 %) 405 nm laser and detecting at two emission ranges of 415-455 and 490-530 nm.

Images were loaded in pairs (the two emission ranges), according to their channel numbers. A sum image was generated, from which the background was subtracted as follows: a normalized Gaussian kernel (with a width of 20 pixels, window size of 121 pixels) was convolved to the image, and the result was subtracted as background. All negative pixel values were set to zero. The resulting image was smoothed again using a local weighted average, and the weight function was a Gaussian with a width of 2 pixels (window size 7 pixels). Next, a threshold was estimated using the 0.9 – 0.95 quantile of the corrected image, and a binary object mask was formed from pixels above this threshold.

The original images were also smoothed using the Gaussian weight function, and the polarization was calculated as: $GP = (I_1 - I_2)/(I_1 + I_2)$ where I_1 is the image measured at 415 – 445 nm, I_2 is the image measured at 490 – 530 nm wavelengths. After comparing the polarization of images measured using 5% Laurdan solution in methanol and the fluorescence spectrum determined in a spectrophotometer, we obtained values of -0.98 and -0.96 respectively, indicating no necessity to use a correction multiplier between the images.
Pixels outside of the object areas defined by the mask image were set to zero, and pixels within the object mask were used to generate histograms typically with 250 bins between -1 and 1.
The above processing was performed using a custom-made python script (available upon request), based on numpy, matplotlib and ImageP packages.
Histograms were fitted to a Gaussian function, or the linear combination of two Gaussian functions in R.

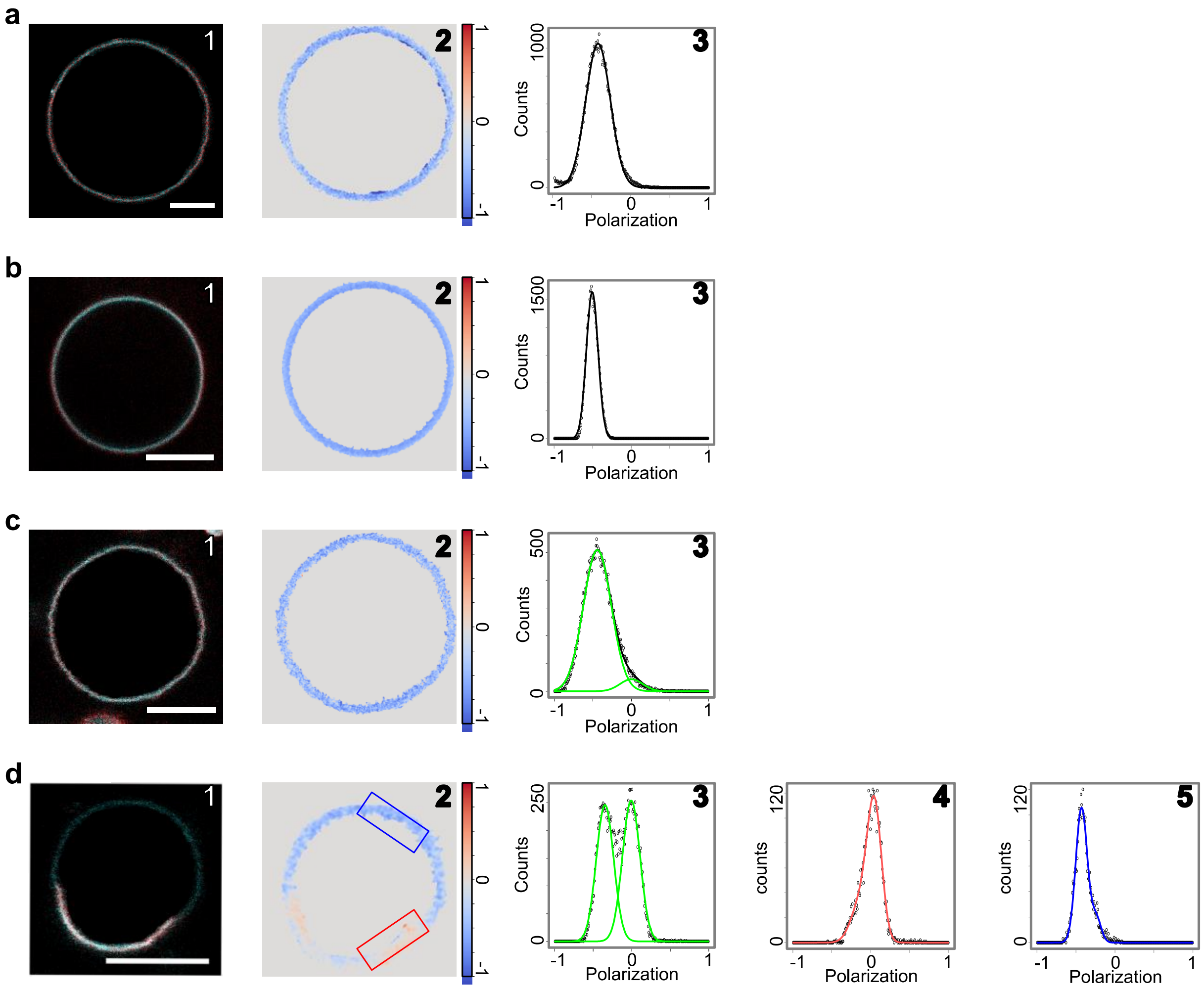

Figure S20. (1) Merged CLSM images of Laurdan emission detected at λ = 415-445 nm (red) and λ = 490-530 nm (cyan). Scale bars: 5 µm (2) Generalized polarization (GP) analysis of (1). (3) Distribution of the GP of Laurdan on the entire membrane. (4-5) Distribution of the GP of Laurdan in the boxes depicted in d2. (a) pure i-combisomes $DP_{85}N_{43}DS_{100}$, (b) i-combisomes with 20 mol% DLPC (C12), (c) i-combisomes with 20 mol% DPPC (C16), (d) i-combisomes with 20 mol% DSPC (C18).

Co-assembly with structure directing glycolipids

We examined the co-assembly of i-CPs and glycolipids. Glycans are the third alphabet of life, and their 3D organization at the cell membrane is of pivotal importance. Figure S21 shows the formation of vesicles by the co-assembly of $DP_{30}N_{30}DS_{100}$ with 20 mol% 16:0 18:1 DG, a lipid containing glucose residue. Almost every glyco–combisomes appeared as a dense sphere with a multitude of layers. Closer analysis evidence the formation of onion vesicles and oligovesicles with high correlation between their bilayer. This correlation may be caused by dipole interactions between the sugar residues that keep the layers together during the process of thin film rehydration. The same behavior has been observed for glycol-dendrimersomes previously.[19]

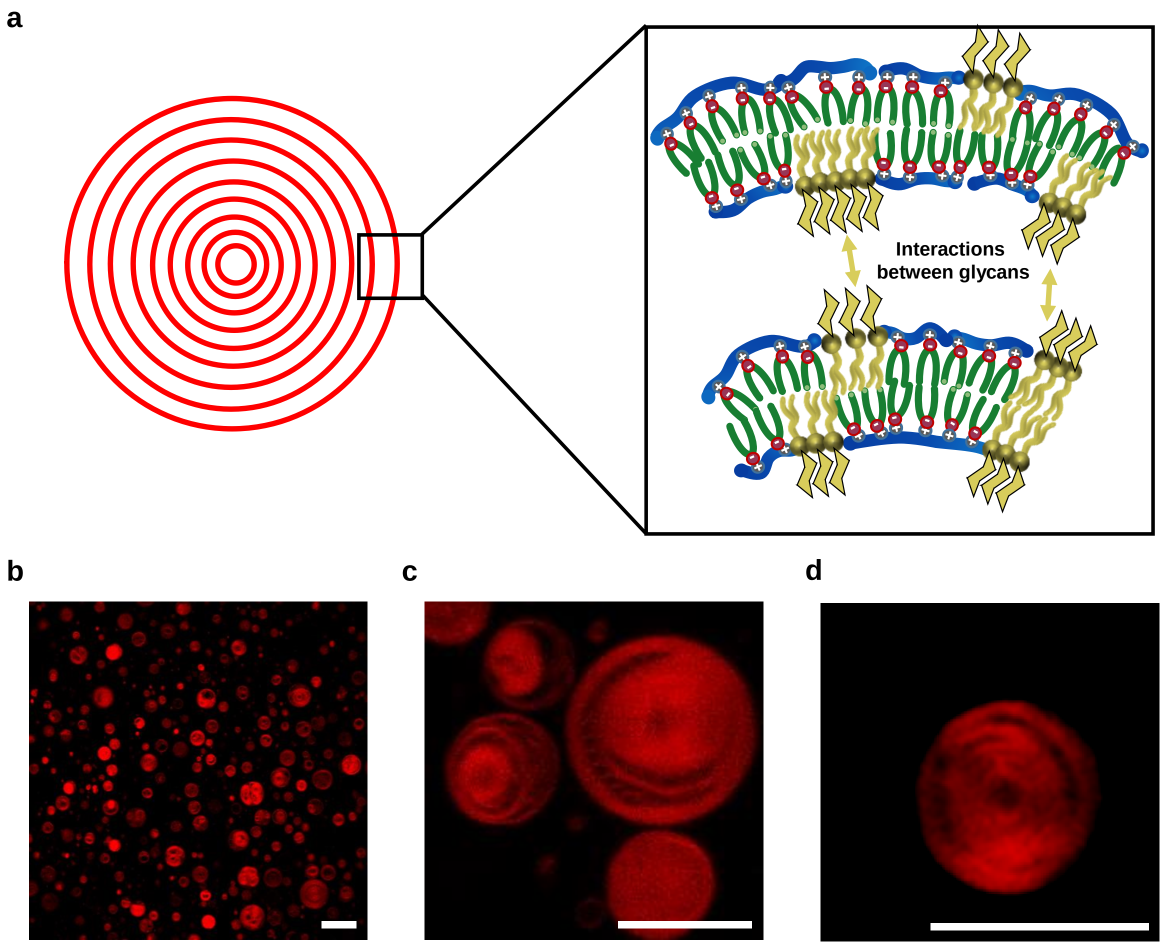

Figure S21. Self-assembly of hybrid i-combisome-glycolipid vesicles. (a) Schematic representation of interbilayer interactions driven by sugar moieties resulting in onion-type vesicles. (b) CLSM overview of hybrid vesicles ($DP_{30}N_{30}DS_{100}$ with 20 mol% 16:0 18:1 DG glucose lipid), Scale: 25 µm, examples of oligovesicular (b) and onion-like vesicles (c). Scale: 10 µm.

Loading of the i-combisomes with nucleic acids

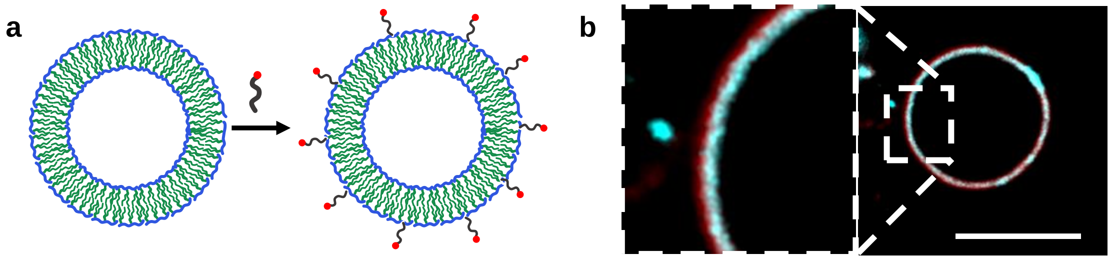

Figure S22: Functionalization of i-combisomes with labeled single-strand DNA. (a) Schematic representation of functionalization and (b) CLSM image of i-combisome (cyan) with homogeneous functionalization with DNA (red).

2.5 Fusion with liposomes

To study the charged and neutral fusion of differently labeled vesicle, we first had to verify that irradiation with the chosen wavelengths specifically excited only the respective fluorescent labels of the liposomes and the i-combisomes. In both cases, we observed no fluorescence when we used the excitation wavelength of the other system (Figure S23). These results indicate that no cross-talk occurs between the fluorescent probes.

Figure S24 shows the fusion of an oligovesicular liposome and a i-combisome. While it was possible to see the colocalization of the dyes in the outer membrane of the resulting fused vesicle, no red fluorescence was observed in the membranes of the internal vesicles. This confirms that only fusion was present and not engulfment.

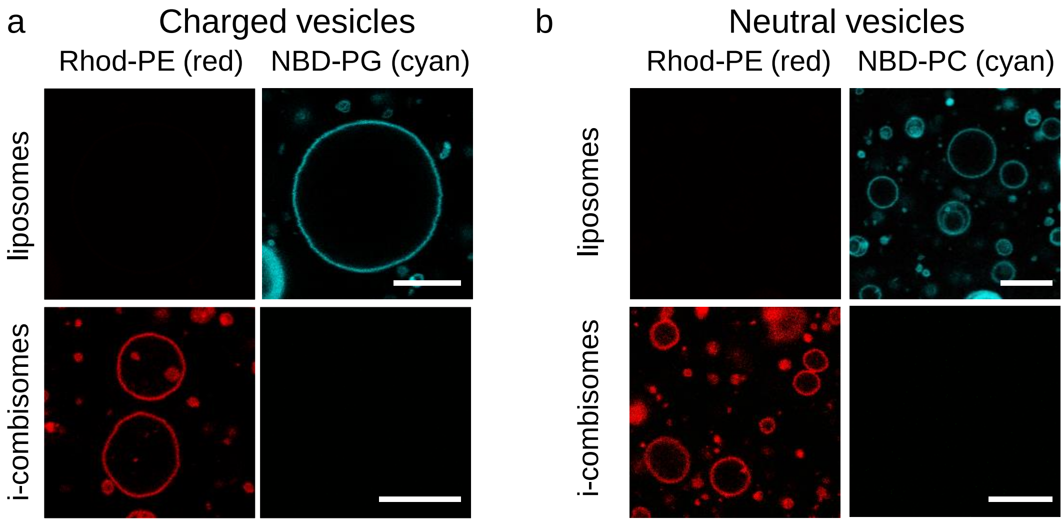

Figure S23: Control experiments to rule out cross-contamination of Rhod and NBD dyes used in fusion experiments for the (a) charged and (b) neutral fusion of i-combisomes with liposomes. Scale bars: 10 µm.

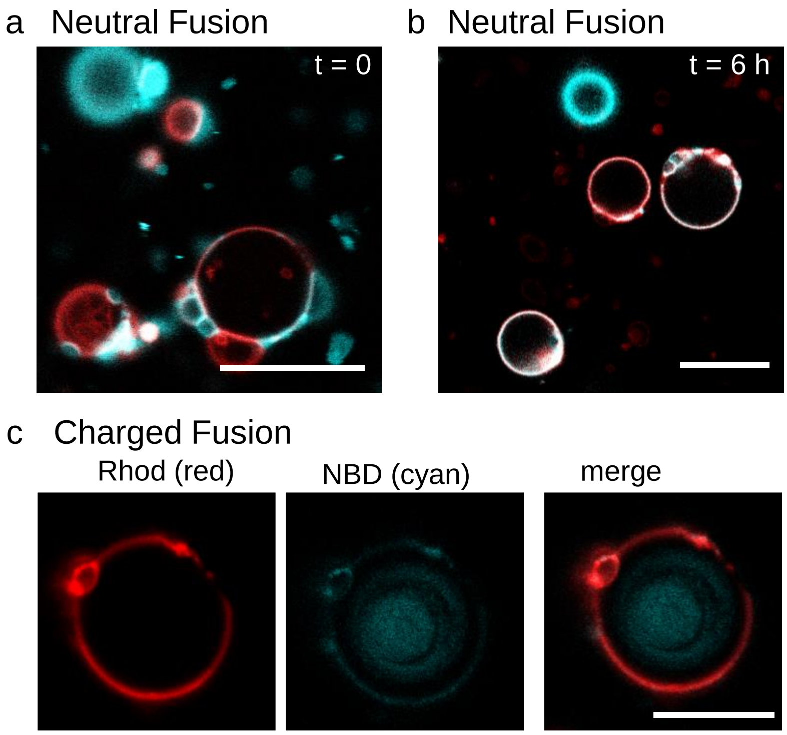

Figure S24: Fusion experiments of liposomes and i-combisomes. Neutral fusion shows patch formation immediately after mixing (a), while after 6 h (b) homogeneous mixing was observed. (c) Mixing of i-combisomes and oligovesicular liposomes (charged) supports a fusion over an engulfment mechanism. Scale bars: 10 µm.

Prototissue assembly

Building synthetic prototissues may serve as simple models to understand emergent collective responses, as advanced biosensors and model diseases. Here we developed a methodology to fabricate a basic prototissue based on i-combisomes embedded surrogate of extracellular matrix. Fibrin network was grown by polymerizing fibrinogen catalyzed by surface-bound thrombin[20] in the presence of i-combisomes of $DP_{85}N_{43}DS_{100}$. Thrombin cleaves fibrinogen into fibrinopeptides that form supramolecular fibers and a network bound to the substrate.[20] Figure S27 shows the fibrin network (labeled blue) encasing a multitude of intact i-combisomes. A closer examination (insets) shows that the i-combisomes are bound to the fibrin bundles in a focal-adhesion-like fashion. This special adhesion is driven by the amphiphilic nature of the fibrinopeptides that form the fibrin fibers. The supramolecular linkage of the i-combisomes to the fibrin matrix allows transferring forces and loads from the matrix to the i-combisomes and opens the door to mimic simple mechanotransduction effects in synthetics cells.

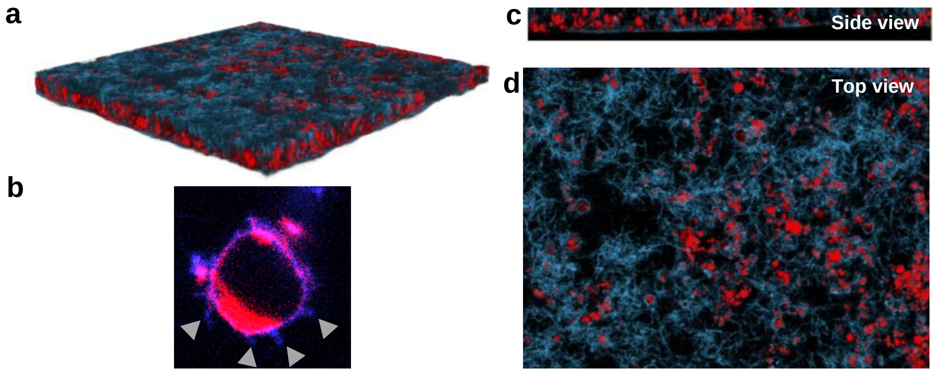

Figure S27. Prototissue formation by embedding i-combisomes (red) in a fibrin-based network (blue). (a) 3D representation of a z-axis scan of the prototissue in CLSM and (c,d) the corresponding side and top view. (b) Shows an embedded i-combisome with focal adhesion points to the matrix (grey arrows).

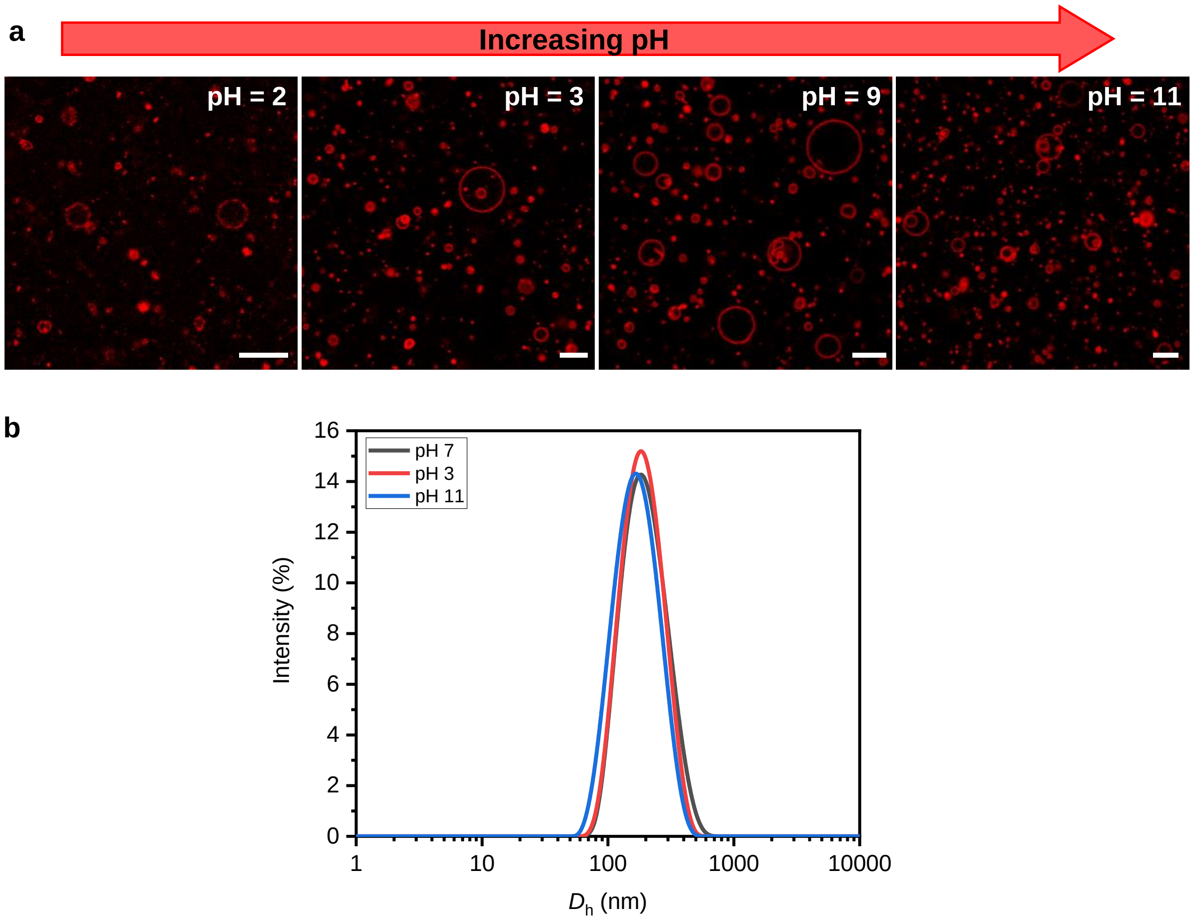

Figure S28. Stability of i-combisomes at different pH. (a) CLSM images of i-combisomes at pH ranging from highly acidic (pH 2-3) to highly alkaline (pH 9-11). Scale bars: 10 µm. (b) Hydrodynamic diameter determined by dynamic light scattering of i-combisomes ($DP_{30}N_{50}DS_{100}$) prepared by injection method in water in neutral (pH = 7) followed by acidification (pH = 3) and alkalization (pH = 11). The vesicles remain unchanged in the range of pH = 3−11.

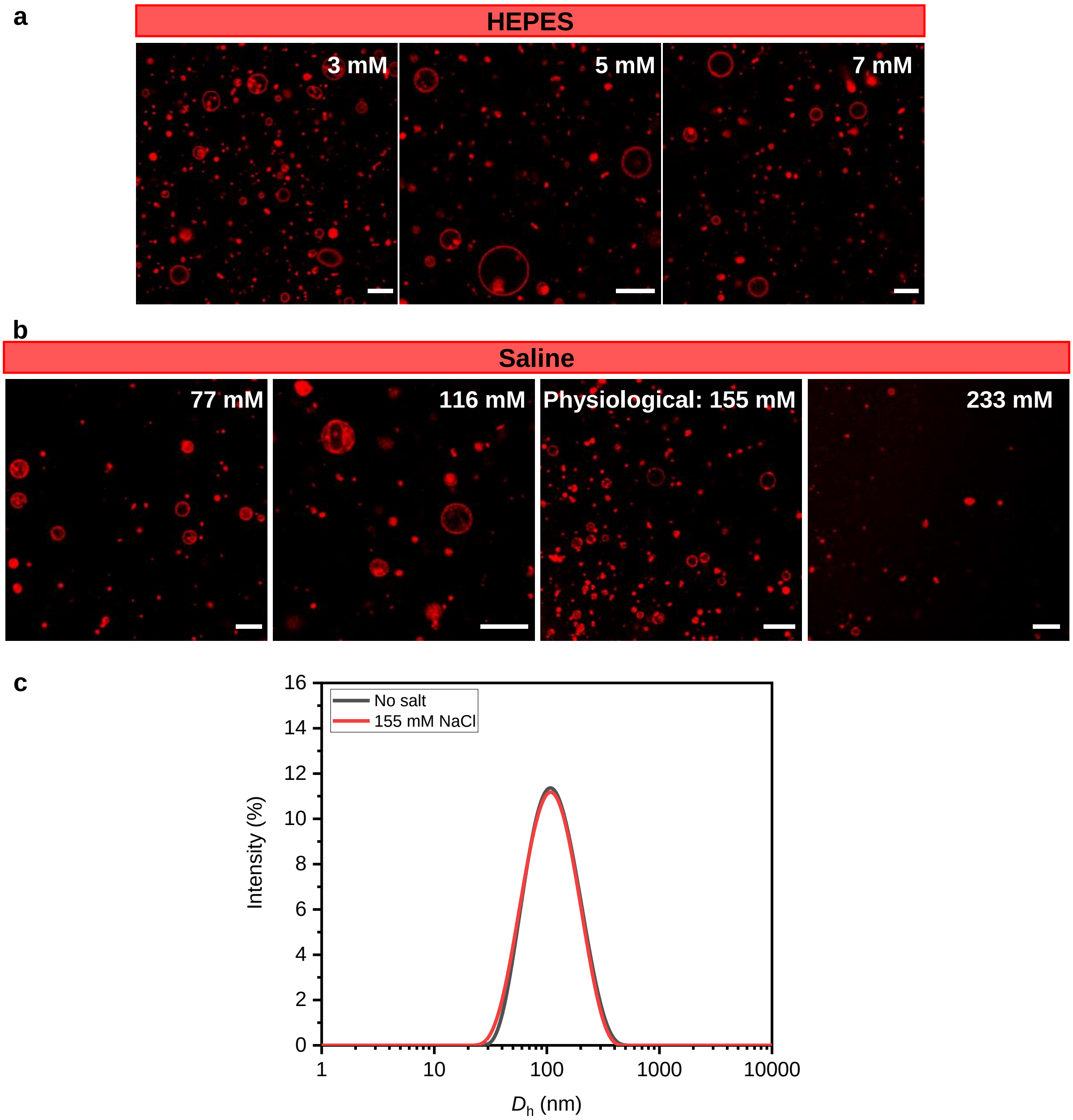

Figure S29. Stability of i-combisomes at different salt concentrations in (a) HEPES buffer ranging from 3-7 mM and (b) Saline solution ranging from 77-233 mM. The i-combisome remained stable upto 155 mM (0.9 w%), afterward the vesicle quality degraded. Scale bars: 10 µm. (c) Hydrodynamic diameter determined by dynamic light scattering of i-combisomes ($DP_{30}N_{50}DS_{100}$) prepared by injection method in water and after mixing with NaCl solution (155 mM). i-Combisomes remain stable up to physiological salt concentration of 155 mM.

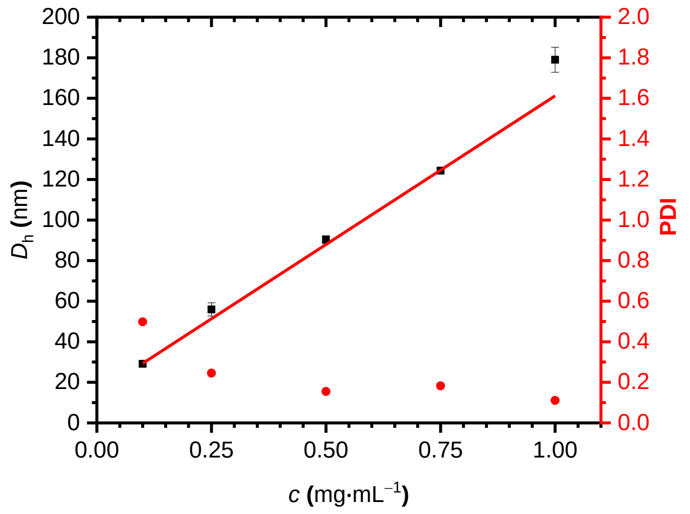

Figure S30. Self-assembly of i-combisomes ($DP_{30}N_{50}DS_{100}$) by injection method at different concentrations. Black squares: Hydrodynamic diameter, red dots: polydispersity index. Both were determined by dynamic light scattering as the average of 3 independent samples.

[1] C. Rodriguez-Emmenegger, E. Brynda, T. Riedel, M. Houska, V. Šubr, A. B. Alles, E. Hasan, J. E. Gautrot, W. T. S. Huck, *Macromol. Rapid Commun.* **2011**, *32*, 952-957.

[2] G. R. Fulmer, A. J. M. Miller, N. H. Sherden, H. E. Gottlieb, A. Nudelman, B. M. Stoltz, J. E. Bercaw, K. I. Goldberg, *Organometallics* **2010**, *29*, 2176-2179.

[3] D. Nečas, P. Klapetek, *Open Physics* **2012**, *10*.

[4] M. J. Abraham, T. Murtola, R. Schulz, S. Páll, J. C. Smith, B. Hess, E. Lindahl, *SoftwareX* **2015**, *1-2*, 19-25.

[5] G. Bussi, D. Donadio, M. Parrinello, *J Chem Phys* **2007**, *126*, 014101.

[6] S. Grimme, J. Antony, S. Ehrlich, H. Krieg, *J Chem Phys* **2010**, *132*, 154104.

[7] B. Hess, *J Chem Theory Comput* **2008**, *4*, 116-122.

[8] G. A. Kaminski, R. A. Friesner, J. Tirado-Rives, W. L. Jorgensen, *The Journal of Physical Chemistry B* **2001**, *105*, 6474-6487.

[9] H. J. C. Berendsen, J. R. Grigera, T. P. Straatsma, *J Phys Chem-Us* **1987**, *91*, 6269-6271.

[10] R. B. Lira, J. Steinkühler, R. L. Knorr, R. Dimova, K. A. Riske, *Scientific Reports* **2016**, *6*, 25254.

[11] T. Riedel, E. Brynda, J. E. Dyr, M. Houska, *J Biomed Mater Res A* **2009**, *88*, 437-447.

[12] J. C. M. Lee, M. Santore, F. S. Bates, D. E. Discher, *Macromolecules* **2002**, *35*, 323-326.

[13] a) J. D. Hunter, *Computing in Science & Engineering* **2007**, *9*, 90-95; b) C. R. Harris, K. J. Millman, S. J. van der Walt, R. Gommers, P. Virtanen, D. Cournapeau, E. Wieser, J. Taylor, S. Berg, N. J. Smith, R. Kern, M. Picus, S. Hoyer, M. H. van Kerkwijk, M. Brett, A. Haldane, J. F. Del Rio, M. Wiebe, P. Peterson, P. Gerard-Marchant, K. Sheppard, T. Reddy, W. Weckesser, H. Abbasi, C. Gohlke, T. E. Oliphant, *Nature* **2020**, *585*, 357-362.

[14] C. A. Schneider, W. S. Rasband, K. W. Eliceiri, *Nat Methods* **2012**, *9*, 671-675.

[15] N. Otsu, *IEEE Transactions on Systems, Man, and Cybernetics* **1979**, *9*, 62-66.

[16] H. A. Faizi, C. J. Reeves, V. N. Georgiev, P. M. Vlahovska, R. Dimova, **2020**.

[17] a) H. P. Duwe, E. Sackmann, *Physica A: Statistical Mechanics and its Applications* **1990**, *163*, 410-428; b) R. Dimova, *Adv Colloid Interface Sci* **2014**, *208*, 225-234.

[18] M. C. Blok, E. C. van der Neut-Kok, L. L. van Deenen, J. de Gier, *Biochim Biophys Acta* **1975**, *406*, 187-196.

[19] Q. Xiao, S. Zhang, Z. Wang, S. E. Sherman, R. O. Moussodia, M. Peterca, A. Muncan, D. R. Williams, D. A. Hammer, S. Vertesy, S. Andre, H. J. Gabius, M. L. Klein, V. Percec, *Proc Natl Acad Sci U S A* **2016**, *113*, 1162-1167.

[20] a) T. Riedel, J. Suttnar, E. Brynda, M. Houska, L. Medved, J. E. Dyr, *Blood* **2011**, *117*, 1700-1705; b) J. Chlupac, E. Filova, T. Riedel, M. Houska, E. Brynda, M. Remy-Zolghadri, R. Bareille, P. Fernandez, R. Daculsi, C. Bourget, L. Bordenave, L. Bacakova, *Physiol Res* **2014**, *63*, 167-177.
